# M-PSGP: a momentum-based proximal scaled gradient projection algorithm for nonsmooth optimization with application to image deblurring

**DOI:** 10.3389/fdata.2025.1704189

**Published:** 2025-11-24

**Authors:** Kexin Ning, Qingguo Lü, Xiaofeng Liao

**Affiliations:** College of Computer Science, Chongqing University, Chongqing, China

**Keywords:** momentum acceleration, adaptive step-size, Barzilai–Borwein rules, proximal gradient descent, nonsmooth constrained optimization

## Abstract

In this study, we focus on investigating a nonsmooth convex optimization problem involving the *l*_1_-norm under a non-negative constraint, with the goal of developing an inverse-problem solver for image deblurring. Research focused on solving this problem has garnered extensive attention and has had a significant impact on the field of image processing. However, existing optimization algorithms often suffer from overfitting and slow convergence, particularly when working with ill-conditioned data or noise. To address these challenges, we propose a momentum-based proximal scaled gradient projection (M-PSGP) algorithm. The M-PSGP algorithm, which is based on the proximal operator and scaled gradient projection (SGP) algorithm, integrates an improved Barzilai-Borwein-like step-size selection rule and a unified momentum acceleration framework to achieve a balance between performance optimization and convergence rate. Numerical experiments demonstrate the superiority of the M-PSGP algorithm over several seminal algorithms in image deblurring tasks, highlighting the significance of our improved step-size strategy and momentum-acceleration framework in enhancing convergence properties.

## 1 Introduction

In the field of image deblurring, the solution of inverse problems can usually be attributed to the solution of constrained optimization problems; thus, the development of optimization solvers for inverse problems with constraints has been a subject of intense research interest. Significantly, the constrained optimization problems arising from image restoration tasks, i.e., denoising, inpainting, and deblurring, can be defined as follows (Bonettini et al., 2008):


$$\begin{array}{l} \min f\left(x\right),\text{ s.t. }x\in S, \end{array}$$ (1)

where *f*(*x*) is a convex function measuring the divergence between the restored and measured data. Due to the ill-posedness of the image restoration problem, a composite regularized function, given by the sum of a fit-to-data function and a penalty function, can be considered to achieve a trade-off between data fidelity and iteration convergence (Tang et al., 2023). Due to the sparsity of image issues, the penalty term generally uses a nonsmooth function, i.e., the *l*_1_-norm; then, the regularized optimization problem can be formed as (Malitsky and Mishchenko, 2024):


$$\begin{array}{l} \min f\left(x\right)+g\left(x\right),\text{ s.t. }x\in S, \end{array}$$ (2)

where *f*(*x*) is the discrepancy term, which could be smooth, and *g*(*x*) is the nonsmooth penalty term. To address this regularized and nonsmooth optimization problem, the proximal gradient method (PGM) is regarded as a robust and efficient approach. PGM is particularly effective in large-scale and high-dimensional composite optimization problems, with wide applications in signal processing (Wang, 2025) and image restoration (Liu et al., 2019). To address the composite optimization problem (Equation 2), the PGM combines gradient descent with a proximal operator on the penalty term, as shown as follows (Parikh and Boyd, 2014):


$$\begin{array}{l} \begin{array}{c} y_{k}={\text{prox}}_{\alpha_{k}g}\left(x_{k}-\alpha_{k}\nabla f\left(x_{k}\right)\right), \end{array} \end{array}$$

where α_*k*_ > 0 is the step size for gradient descent, and the proximal operator for the penalty function *g* is defined as follows:


$$\begin{array}{l} \begin{array}{c} {\text{prox}}_{\alpha_{k}g}\left(x\right)=\underset{z\in {\mathbb{R}}^{N}}{\arg\min}\alpha_{k}g\left(z\right)+\frac{1}{2}||x-z|{|}^{2}. \end{array} \end{array}$$

By invoking the proximal operator, the PGM effectively manages non-smooth components and promotes sparse solutions, thus outperforming traditional gradient-based techniques in non-smooth optimization contexts (Aravkin et al., 2022). Classically, the two-step iterative shrinkage thresholding (TwIST) algorithm extends the PGM by introducing a momentum term that relies on the two previous iterations, leading to faster convergence rates for ill-conditioned problems (Bioucas-Dias and Figueiredo, 2007). Thereafter, the fast iterative shrinkage thresholding (FISTA) algorithm was proposed, which improves the PGM by incorporating the Nesterov momentum term and an adaptive step size, thereby accelerating the global convergence rate (Beck and Teboulle, 2009). Moreover, optimization problems in practical applications are often constrained by domain-specific requirements, which motivate the development of the gradient projection method (GPM) to address constrained optimization problems (Zhou et al., 2025). For solving Equation 1, the GPM combines gradient descent with a projection operator, leading to the following iteration:


$$\begin{array}{l} y_{k}=\Pi_{S}\left(x_{k}-\alpha_{k}\nabla f\left(x_{k}\right)\right), \end{array}$$

in which the projection operator of a vector *x* ∈ ℝ^*N*^ onto the region set S is defined as follows:


$$\begin{array}{l} \begin{array}{c} \Pi_{S}\left(x\right)=\underset{z\in S}{\arg\min}\frac{1}{2}||x-z|{|}^{2}. \end{array} \end{array}$$

The projection operation can achieve minimal computational expense while restricting the iterative vector onto the feasible set S. This is evident in the specific case of problem (Equation 2), i.e., lasso regression and ridge regression, where projection can be computed in linear time (Maroni et al., 2025). Consequently, the GPM and its improvements stand as credible schemes to address image deblurring tasks, thanks to their low per-iteration computational overhead (Chen et al., 2025).

Through an optimized fusion of PGM and GPM, a scaled gradient projection (SGP) algorithm was proposed to address the constrained composite problem (Equation 2) (Bonettini and Prato, 2015). The SGP algorithm introduces a scaling matrix *D*_*k*_ during the proximal gradient descent; that is,


$$\begin{array}{l} y_{k}=\Pi_{S,D_{k}}\left[{\text{prox}}_{\alpha_{k}g}^{D_{k}^{-1}}\left(x_{k}-\alpha_{k}D_{k}\nabla f\left(x_{k}\right)\right)\right]. \end{array}$$ (3)

Compared with the FISTA algorithm, SGP demonstrates superior convergence rates and enhances deblurring performance. Recently, aiming to accelerate the solution of the composite optimization method, an optimal accelerated composite optimization (OptISTA) algorithm to solve Equation 2 was developed, which improves FISTA by optimizing the step size via a double-function method and theoretically establishes an exact matching lower bound for composite optimization problems (Jang et al., 2025). Afterwards, the improved OptISTA (IOptISTA) algorithm was proposed, which incorporates a weighting matrix technique and achieves superior numerical performance in image deblurring tasks (Wang et al., 2025).

To accelerate convergence during optimization, the Barzilai-Borwein rules, which serve as adaptive step-size strategies that determine step sizes without relying on online searches, have been widely investigated and improved (He et al., 2023). Serving as an effective and seminal step-size selection strategy, the Barzilai-Borwein (BB) step-size selection rules have garnered significant attention, which approximate the Hessian matrix inverse in a quasi-Newton manner, providing a step size that can adaptively adjust to the descent direction of the problem, which leads to faster convergence rates (Barzilai and Borwein, 1988). Recently, in the context of *l*_1_-regularized problems, a novel step-size selection rule within the proximal gradient framework was proposed, which achieves acceleration by preemptively identifying the optimal redundant components using the spectral properties of the BB step size (Crisci et al., 2024). Beyond step-size improvement, momentum methods provide additional convergence acceleration (Zhang et al., 2023). Classically, the Heavy-Ball (HB) algorithm leverages momentum from past gradients to amplify descent directions, thereby improving convergence rates in ill-conditioned optimization landscapes (Polyak, 1964). Afterwards, Nesterov proposed an Accelerated Gradient (NAG) algorithm, which achieves faster convergence by calculating the gradient at an extrapolated future position based on current momentum (Nesterov, 1983). Building upon foundational momentum techniques, a series of momentum frameworks has been developed to achieve enhanced convergence properties. In Yang et al. (2016), a unified framework was developed to implement a range of momentum acceleration variants by altering related parameters. Recently, another unified paradigm was proposed, which features two momentum-based algorithm variants for decentralized stochastic gradient descent (Du et al., 2024).

Motivated by the effectiveness of the SGP algorithm in combining PGM and GPM and inspired by acceleration strategies involving step-size rules and momentum techniques, we focus on developing a solver for the constrained *l*_1_-regularized optimization problem, aiming to accelerate convergence while preserving optimization performance in image deblurring.

The main contributions in this study are summarized below.

We propose a momentum-based proximal scaled gradient projection (M-PSGP) algorithm that combines the procedure of the SGP algorithm with a unified momentum framework (UM). Numerical experiments demonstrate the effectiveness of UM in stabilizing and accelerating the convergence rate of our algorithm.

• We design a new step-size selection rule BB2^*^ and apply it in the M-PSGP algorithm. The BB2^*^ rule can be regarded as an enhancement of the BB-like step-size rule proposed by Crisci et al. (2024). By combining it with UM, a dual acceleration effect could be achieved. Numerical experiments illustrate the superiority of BB2^*^ by comparing it with other rules in terms of accelerating the convergence rate.

• Compared with other seminal proximal gradient projection algorithms, the M-PSGP algorithm can accelerate the convergence rate of *l*_1_-regularized optimization problems. Meanwhile, the M-PSGP algorithm can achieve better performance in image deblurring tasks, particularly when applied to images generated by convolving a Gaussian blur with originally astronomical or medical images and adding Gaussian white noise.

*Organization:* In Section 2, we describe the constrained *l*_1_-regularized optimization problem by presenting the image formation and the principle of image deblurring. In Section 3, we present the SGP algorithm in detail and propose the M-PSGP algorithm. In Section 4, we perform a convergence analysis for the M-PSGP algorithm. In Section 5, we conduct a series of comparative experiments and demonstrate the effectiveness of the M-PSGP algorithm. In Section 6, our final remarks and future work are given.

*Notation:* Throughout the article, $x={\left(x_{1},\cdots \;,x_{N}\right)}^{\text{T}}\in {\mathbb{R}}^{N}$ is a vector stacked from the two-dimensional original image *X* ∈ ℝ^*N*^, *N* = *n* × *n*. Similarly, the vector $y={\left(y_{1},\cdots \;,y_{N}\right)}^{\text{T}}\in {\mathbb{R}}^{N}$ is stacked from the observed image *Y* ∈ ℝ^*N*^, and the vector $b={\left(b_{1},\cdots \;,b_{N}\right)}^{\text{T}}\in {\mathbb{R}}^{N}$ is stacked from the background additive noise *B* ∈ ℝ^*N*^. *A* ∈ ℝ^*N* × *N*^ is a given matrix modeling the blur effect. The symbol λ is a regularization coefficient. The symbol ${\left||x|\right|}_{1}=\overset{N}{\underset{i=1}{\sum }}|x_{i}|$ is the *l*_1_-norm of *x*. ||*x*|| denotes the standard Euclidean norm for *x*, ${\left||x|\right|}_{D}=\sqrt{x^{\text{T}}Dx}$ indicates the *D*-norm, where *D* is a symmetric positive definite matrix. ∇*f*(*x*) is the gradient of the continuous function *f* at *x*, and ∂*g*(*x*) is the subdifferential of the semicontinuous function *g* at *x*.

## 2 Problem description

### 2.1 Image formation and deblurring modeling

In this work, we note that the image blur is caused by out-of-focus blur during detection. Under the assumption that the blur is uniform across the original image, the blurring process can be modeled as a convolution process:


$$\begin{array}{l} Y=A⊗X+B, \end{array}$$

where *A* is the blur kernel, *Y* is the blurred image, *X* is the original image, and *B* represents background additive noise.

The goal of image deblurring is to recover the original image *X* from the observed *Y*. Due to the extreme ill-conditioning of *A*, the original image cannot be directly obtained through matrix operations. Hence, we employ the Bayesian approach, which uses prior information about the original object, to model the solution process of this inverse problem. The Bayes formula provides the conditional probability of *x*_*i*_ given the value *y*_*i*_:


$$\begin{array}{l} P_{X}\left(x_{i}|y_{i}\right)=\frac{P_{Y}\left(y_{i}|x_{i}\right)P_{X}\left(x_{i}\right)}{P_{Y}\left(y_{i}\right)}. \end{array}$$

Assume that the blur kernel *A* and the background noise *B* follow Gaussian distributions; then, the distribution of blurred pixel values *y*_*i*_ is given by the following:


$$\begin{array}{l} y_{i}\sim N\left(Ax_{i}+b_{i},\sigma^{2}\right), \end{array}$$

where σ is the standard deviation of *B*. Then, the likelihood is estimated as follows:


$$\begin{array}{l} \begin{array}{c} P_{Y}\left(y|x\right)=\overset{N}{\underset{i=1}{\prod }}P_{Y}\left(y_{i}|x_{i}\right)=\overset{N}{\underset{i=1}{\prod }}\frac{1}{\sqrt{2\pi \sigma^{2}}}\exp\left(-\frac{{\left(y_{i}-\left(Ax_{i}+b_{i}\right)\right)}^{2}}{2\sigma^{2}}\right). \end{array} \end{array}$$ (4)

Assume that *X* is a Gibbs random field with a distribution:


$$\begin{array}{l} P_{X}\left(x\right)=\overset{N}{\underset{i=1}{\prod }}P_{X}\left(x_{i}\right)=\overset{N}{\underset{i=1}{\prod }}\frac{1}{Z}\exp\left(-\lambda g\left(x_{i}\right)\right), \end{array}$$

where *Z* is a normalization constant, and *g*(*x*) is a given function, usually called the penalization function. By taking the negative logarithm of *P*_*Y*_(*y*_*i*_|*x*_*i*_)*P*_*X*_(*x*_*i*_) and considering the maximum estimation of the posterior probability distribution, the image deblurring can be modeled as an optimization problem of the form:


$$\begin{array}{l} \min F\left(x\right)=f\left(x\right)+\lambda g\left(x\right), \end{array}$$ (5)

in which *f*(*x*) is the simplified negative logarithm of Equation 4 defined as follows:


$$\begin{array}{l} f\left(x\right)=\overset{N}{\underset{i=1}{\sum }}{\left(y_{i}-\left(Ax_{i}+b_{i}\right)\right)}^{2}. \end{array}$$ (6)

### 2.2 Problem formulation

Let S ⊆ ℝ^*N*^ be a closed convex set. For arbitrary *x* ∈ S, *f*(*x*): S → ℝ is a convex, continuously differentiable function and *g*(*x*): S → ℝ is a convex and lower semicontinuous function. Following Equation 5, we consider the constrained optimization problem as follows:


$$\begin{array}{l} \begin{array}{cc} \min\;F\left(x\right)=\frac{1}{2}||Ax-y|{|}^{2}+\lambda ||x|{|}_{1},\\ \text{s.t.} & x\geq 0, \end{array} \end{array}$$ (7)

in which, under the assumption that the blur kernel and background noise follow Gaussian distributions, following estimation (Equation 4) and its simplified negative logarithm (Equation 6), we let *f*(*x*) be a least squares function and choose the *l*_1_-norm to serve as *g*(*x*). Under these conditions, *F*(*x*) is convex and the gradient ∇*f*(*x*) = *A*^⊤^(*Ax* − *b*), which implies that for any *x, y* ∈ S:


$$\begin{array}{l} ||\nabla f\left(x\right)-\nabla f\left(y\right)|{|}_{2}=||A^{\text{T}}A|{|}_{2}||x-y|{|}_{2}. \end{array}$$ (8)

Thus, ∇*f*(**x**) is *L*-Lipschitz continuous with $L=||{\text{A}}^{⊤}\text{A}|{|}_{2}$.

In the following, we will provide some related definitions and basic properties.

Definition 1 (Stationary point). Given a closed convex set S ⊆ ℝ^*N*^ and a proper, convex function *F*: S → ℝ, a point x^*^ ∈ S is the stationary point of *F* if 0 ∈ ∂*F*(x^*^).

Below, the definition of subdifferential for a general function is provided.

Definition 2 (Limiting subdifferential). Given a closed convex set S ⊆ ℝ^*N*^ and a convex and lower semicontinuous function *g*: S → ℝ, the limiting subdifferential at a point *x* ∈ S is:


$$\begin{array}{l} \begin{array}{c} \partial g\left(x\right)=\left\{w\in S:\forall z\in S,g\left(z\right)\geq g\left(x\right)+{\left(z-x\right)}^{\text{T}}w\right\}. \end{array} \end{array}$$

In the situation, the subdifferential of *g*(*x*) = λ||*x*||_1_ can be computed by:


$$\begin{array}{l} \begin{array}{c} \partial \left(\lambda {\left||x|\right|}_{1}\right)=\{\begin{array}{cc} \lambda \text{sign}\left(x\right), & if\;x\neq 0,\\ \left[-\lambda ,\lambda \right], & if\;x=0. \end{array} \end{array} \end{array}$$

In our study, the proximal operator is applied based on the definition 3.

Definition 3 (Proximal operator). Given a closed convex set S ⊆ ℝ^*N*^ and a threshold L > 1, let $D_{L}=\left\{D\in R^{N\times N}|D>0,||D||\leq L,||D^{-1}||\leq L\right\}$, the proximal operator ${\text{prox}}_{\alpha g}^{D}:S\to {\mathbb{R}}^{N}$ associated with a non-negative parameter α is defined as:


$$\begin{array}{l} {\text{prox}}_{\alpha g}^{D^{-1}}\left(x\right)=\underset{z\in S}{\arg\min}\left[\alpha g\left(z\right)+\frac{1}{2}||x-z|{|}_{D}\right]. \end{array}$$ (9)

The Lipschitz continuity property of the operator (Equation 9) is stated in Lemma 1:

Lemma 1. (Bauschke and Combettes, 2017) If *g*: S → ℝ is convex, proper and lower semicontinuous, for any *x, y* ∈ S, it satisfies:


$$\begin{array}{l} ||{\text{prox}}_{\alpha g}^{D^{-1}}\left(x\right)-{\text{prox}}_{\alpha g}^{D^{-1}}\left(y\right)||\leq ||x-y|{|}_{D}. \end{array}$$

Definition 4 (Projection operator). Given a closed convex set S ⊆ ℝ^*N*^ and a threshold *L* > 1, let $D_{L}=\left\{D\in R^{N\times N}|D>0,||D||\leq L,||D^{-1}||\leq L\right\}$, the projection operator $\Pi_{S,D}:S\to {\mathbb{R}}^{N}$ is defined as follows:


$$\begin{array}{l} \Pi_{S,D}=\underset{z\in S}{\arg\min}\frac{1}{2}||x-z|{|}_{D}^{2}. \end{array}$$ (10)

The Lipschitz continuity property of the operator (Equation 10) is stated as Lemma 2:

Lemma 2. (Bonettini et al., 2008) If *D* ∈ *D*_*L*_ and *x, y* ∈ S, then:


$$\begin{array}{l} \left(\text{i}\right)||\Pi_{S,D}\left(x\right)-\Pi_{S,D}\left(y\right)||\leq L^{2}||x-y||\\ \left(\text{ii}\right)\langle \Pi_{S,D}\left(x\right)-x,\Pi_{S,D}\left(x\right)-y\rangle \leq 0. \end{array}$$

Naturally, corresponding to the progress of gradient descent, the stationary point can be confirmed by the iterative value, as in Lemma 3:

Lemma 3. (Bonettini and Prato, 2015) A vector x^*^ ∈ S is a stationary point of Equation 7 if and only if


$$\begin{array}{l} x^{*}=\Pi_{S}\left[{\text{prox}}_{\alpha \lambda ||\cdot |{|}_{1}}^{D^{-1}}\left(x^{*}-\alpha D\nabla f\left(x^{*}\right)\right)\right]. \end{array}$$

## 3 Methods

### 3.1 The SGP algorithm

The SGP algorithm (Bonettini and Prato, 2015) combines the scaled gradient descent method (Equation 3) with a line search procedure, resulting in the following iteration:


$$\begin{array}{l} y_{k}=\Pi_{S,D_{k}}\left[{\text{prox}}_{\alpha_{k}g}^{D_{k}^{-1}}\left(x_{k}-\alpha_{k}D_{k}\nabla f\left(x_{k}\right)\right)\right], \end{array}$$ (11)

$$\begin{array}{l} x_{k+1}=x_{k}+\lambda_{k}d_{k}. \end{array}$$ (12)

It employs a symmetric positive definite matrix *D*_*k*_ in front of ∇*f*(*x*^*k*^) during the process of gradient descent, and the entries $d_{i}^{k}$ in $D_{k}=\text{diag}\left(d_{1}^{k},d_{2}^{k},\cdots \;,d_{N}^{k}\right)$ are defined as:


$$\begin{array}{l} d_{i}^{k}=\min\left\{L,\max\left\{\frac{1}{L},x_{i}^{k}\right\}\right\}, \end{array}$$ (13)

where *L* is an appropriate threshold, customarily chosen as the Lipschitz constant of the objective function. Based on the BB rules, α_*k*_ is confirmed as follows:


$$\begin{array}{l} \alpha_{k}^{\text{BB}1}=\frac{s_{k-1}^{\text{T}}D_{k}^{-1}D_{k}^{-1}s_{k-1}}{s_{k-1}^{\text{T}}D_{k}^{-1}z_{k-1}}, \end{array}$$ (14)

$$\begin{array}{l} \alpha_{k}^{\text{BB}2}=\frac{s_{k-1}^{\text{T}}D_{k}z_{k-1}}{z_{k-1}^{\text{T}}D_{k}D_{k}z_{k-1}}, \end{array}$$ (15)

where *s*_*k*−1_ = *x*_*k*_ − *x*_*k*−1_ and *z*_*k*−1_ = ∇*f*(*x*_*k*_)−∇*f*(*x*_*k*−1_). Further, the step-size α_*k*_ in Equation 12 is adaptively alternated based on $\alpha_{k}^{\text{BB}1}$ or $\alpha_{k}^{\text{BB}2}$.

Specifically, if the iterate *y*^*k*^ obtained from the projection is identical to the current iterate *x*^*k*^, then *x*^*k*^ is recognized as a stationary point. If *y*^*k*^ ≠ *x*^*k*^, a descent direction is defined as *d*_*k*_ = *y*_*k*_ − *x*_*k*_, and it is incorporated into the update procedure specified in Equation 12, where λ_*k*_ is the step-size for the descent iteration.

### 3.2 The proposed M-PSGP algorithm: momentum-based proximal scaled gradient projection algorithm

The update direction of PGM is solely determined by the negative gradient of the continuous part of the objective function, which could lead to oscillations during the iteration. The SGP invokes the scaled matrix *D*_*k*_ before the negative gradient, which enhances the descent step size but also raises the oscillations. To address such circumstances, momentum methods can mitigate oscillations by incorporating a velocity term that averages past gradients, thereby leveraging inertia to stabilize direction in the face of perturbations (Xie et al., 2025). Moreover, by leveraging historical gradient information, the momentum method could maintain large updates even in low-curvature areas, indicating the acceleration effect on the convergence rate (Loizou and Richtárik, 2020).

Considering the effects of acceleration and stability on momentum methods, we integrate a unified momentum framework, UM (Yang et al., 2016), within SGP, resulting in the M-PSGP algorithm for the problem (Equation 7). Setting α > 0, β ∈ [0, 1], and *s* ≥ 0, the update process of UM is described as follows:


$$\begin{array}{l} \begin{array}{l} \text{UM}:\{\begin{array}{c} \begin{array}{c} y_{k}=x_{k}-\alpha \nabla f\left(x_{k}\right),\\ y_{k}^{s}=x_{k}-s\alpha \nabla f\left(x_{k}\right),\\ x_{k+1}=y_{k}+\eta \left(y_{k}^{s}-y_{k-1}^{s}\right). \end{array} \end{array} \end{array} \end{array}$$ (16)

• When *s* = 0, the update is reduced to the HB method:


$$\begin{array}{l} \begin{array}{c} x_{k+1}=x_{k}-\alpha \nabla f\left(x_{k}\right)+\eta \left(x_{k}-x_{k-1}\right). \end{array} \end{array}$$ (17)

• When *s* = 1, the update is exactly the form of the NAG method:


$$\begin{array}{l} \begin{array}{c} \{\begin{array}{l} \begin{array}{c} y_{k}=x_{k-1}-\alpha \nabla f\left(x_{k-1}\right),\\ x_{k+1}=y_{k}+\eta \left(y_{k}-y_{k-1}\right), \end{array} \end{array} \end{array} \end{array}$$ (18)

• When $s=\frac{1}{1-\eta }$, the update of *x*_*k*+1_ is essentially a gradient method (GM) to achieve acceleration:


$$\begin{array}{l} \begin{array}{c} x_{k+1}=x_{k}-\frac{\alpha }{1-\eta }\left(\nabla f\left(x_{k}\right)-\nabla f\left(x_{k-1}\right)\right)+\eta \left(x_{k}-x_{k-1}\right), \end{array} \end{array}$$ (19)

**Figure 1:**
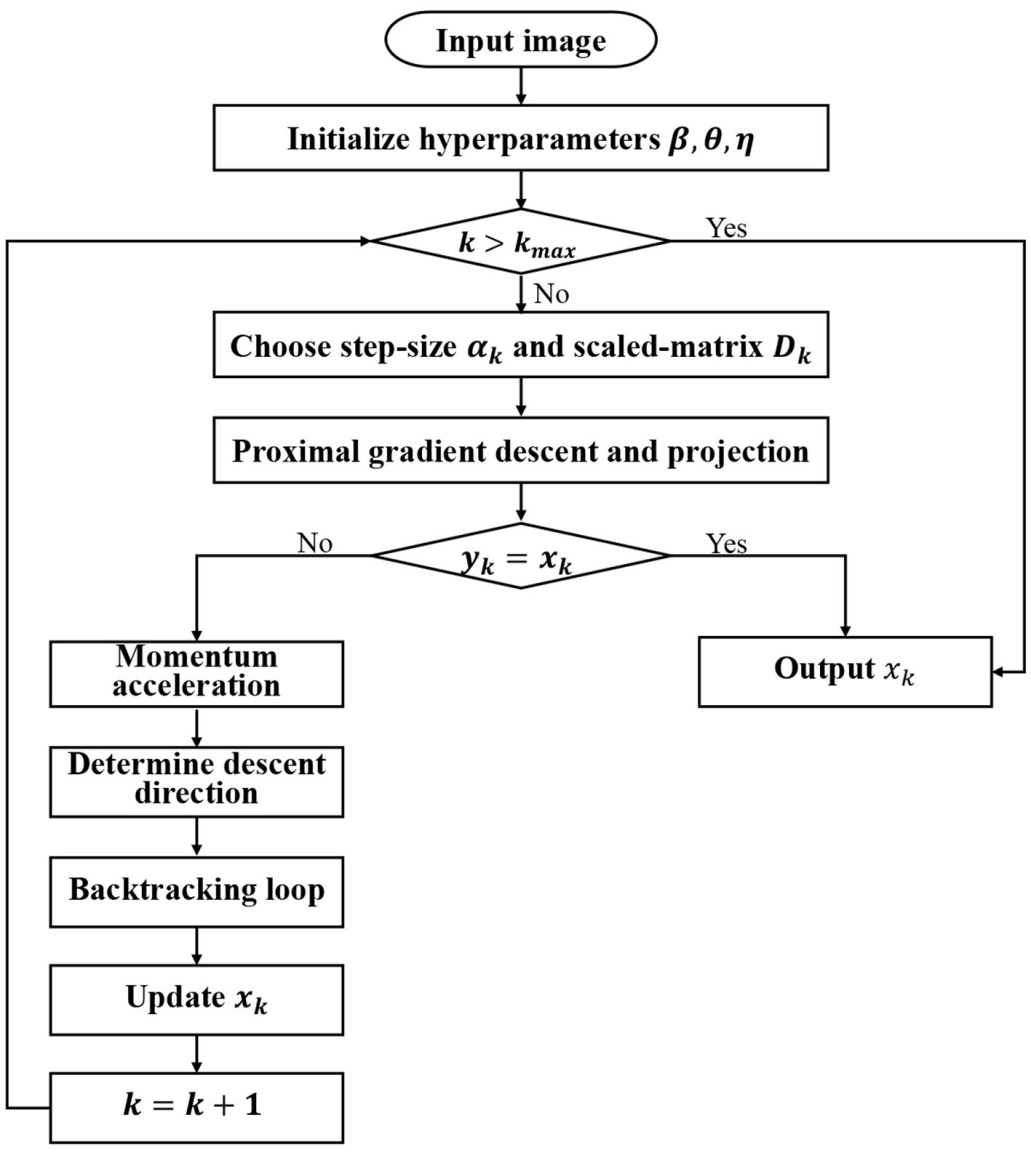
The flow diagram of the Algorithm 1.

By incorporating the UM (Equation 16) into the procedure (Equations 11, 12), the M-PSGP algorithm is delineated in Algorithm 1. For clarity, we also provide the exact flow diagram of Algorithm 1 in Figure 1.

Algorithm 1 **M-PSGP.**

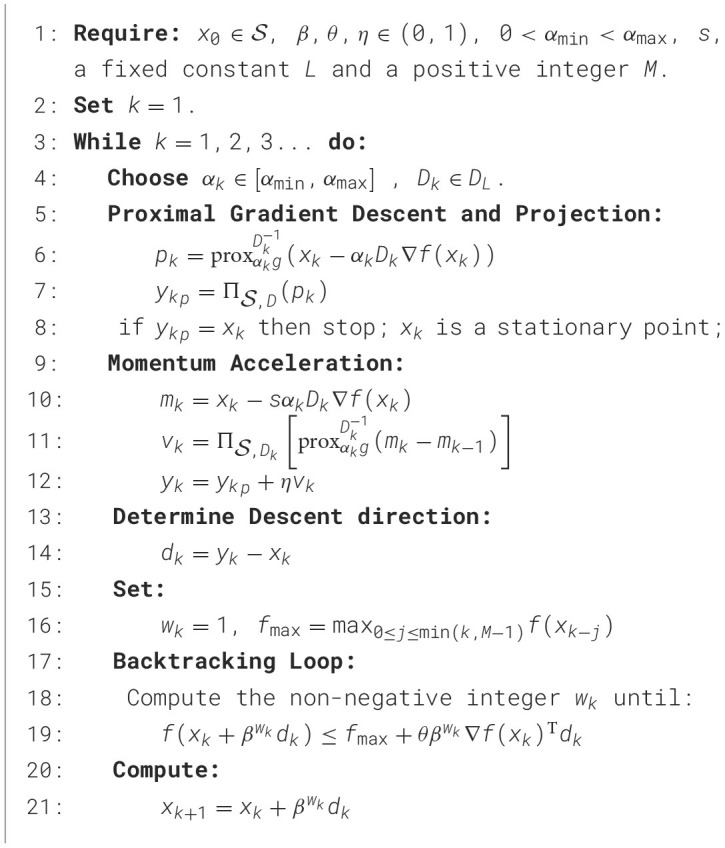

As illustrated in Figure 1, beyond parameter initialization, the algorithm primarily consists of four key computational phases, including proximal gradient descent and projection, momentum acceleration, determining the descent direction, and the backtracking loop. In the **proximal gradient descent and projection** step, the M-PSGP computes a gradient descent step on the smooth component *f*(*x*), then applies the proximal operator to account for the non-smooth regularizer *g*(*x*) in line 6. Subsequently, line 7 applies the projection operator to ensure constraint satisfaction. In the **momentum acceleration** step, the M-PSGP first computes the next intermediate position *m*_*k*_, as shown in line 10. The calculation of momentum *v*_*k*_ is defined as *m*_*k*_ − *m*_*k*−1_, and to satisfy the non-negative constraint, it should be projected as in line 11. The value *v*_*k*_ captures the change between successive intermediate positions during the optimization process. Thus, in line 12, the M-PSGP incorporates information about the past gradients into the update step, which helps accelerate and stabilize convergence toward the stationary point. Here, η is a hyperparameter that modulates the effect of momentum on the update direction. Subsequently, the **descent direction** is formally defined as the vector difference between the current iterate and the momentum-corrected candidate point in line 14. Finally, a **backtracking line search loop** in line 19 is executed to find an appropriate step size along this direction, which recalls the generalized non-monotone Armijo-type line search proposed by Zhang (2010).

**Figure 2:**
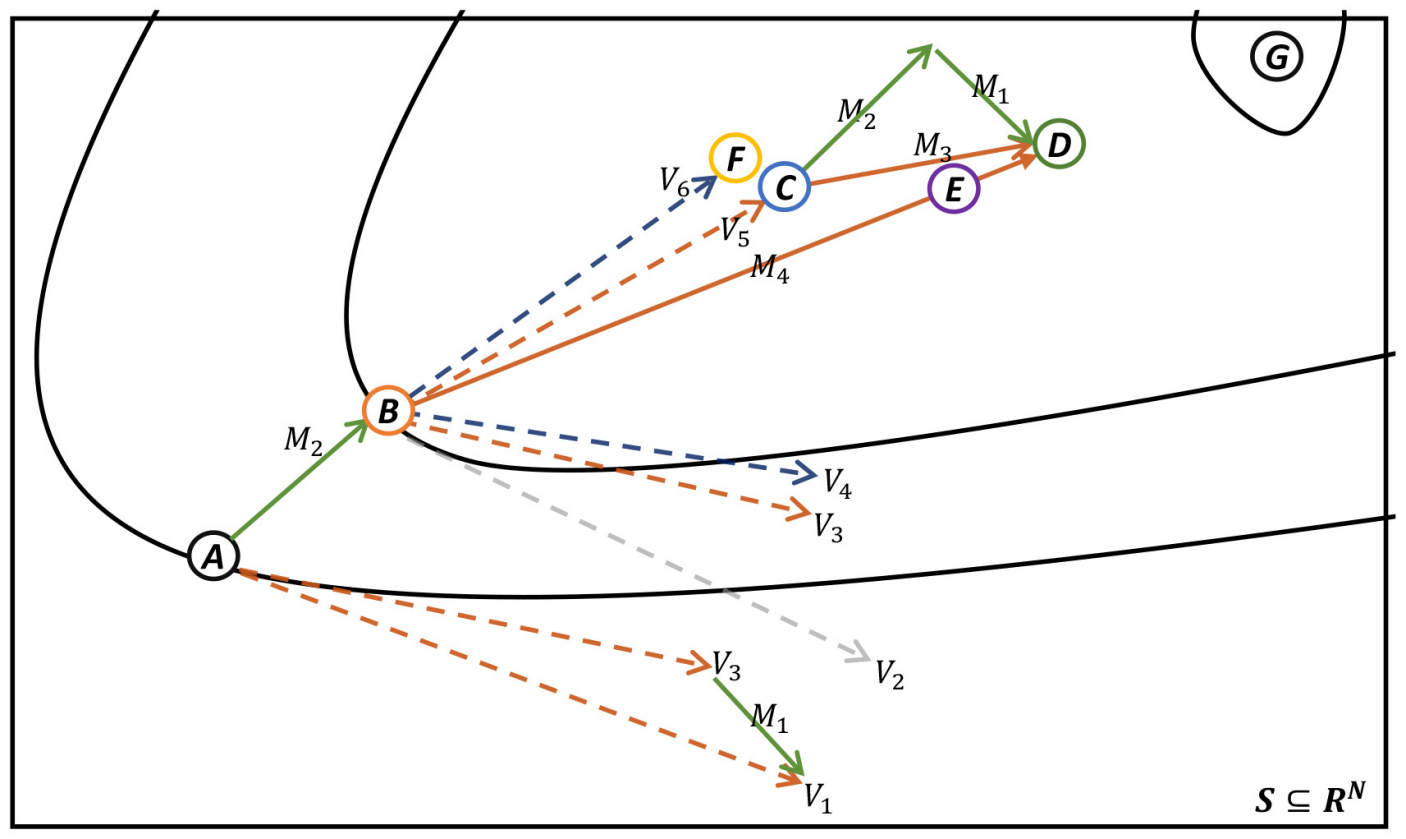
Iteration process of M-PSGP (accelerated by NAG) and SGP Algorithms. Nodes and vectors are noted in Table 1.

In Figure 2, we present a schematic diagram of a single iteration for both the M-PSGP and SGP algorithms. To provide a more intuitive explanation, we present Table 1, which specifies the definitions of all nodes and vectors, along with their correspondences to the key steps of Algorithm 1. Based on the explanations provided in Table 1, we can conclude from Figure 2 that, under identical initial conditions, the *E* node (obtained by the M-PSGP algorithm) moves closer to the stationary point of *F*(*x*) after one iteration than the *F* node (obtained by the SGP algorithm). This observation further verifies, from a vector operation perspective, the acceleration effect of the M-PSGP algorithm. Additionally, it should be noted that, for the sake of clarity and conciseness, we have omitted explicit emphasis on projection operations, as all vectors are assumed to be constrained within the feasible domain by default. Likewise, nodes *C* and vector *M*_3_ are also presented as the results obtained after applying the proximal operator.

**Table 1:** Notation of nodes and vectors in Figure 2.

**Type**	**Label**	**Meaning**	
Node	*A*	*x*_*k*−1_	
	*B*	*x*_*k*_
	*C*	*y*_*k*_*p*__	Within line 7 of Algorithm 1
	*D*	*y*_*k*_	Within line 12 of Algorithm 1
	**E**	***x*_*k*+1_**	**the** **(*k* + 1)-th iteration value of M-PSGP**
	**F**	${\tilde{x}}_{k+1}$	**the** **(*k* + 1)-th iteration value of SGP**
	*G*	*x* ^⋆^	The stationary point of *F*(*x*)
Vector	*V*_1_	α_*k*−1_*D*_*k*−1_ ∇ *f*(*x*_*k*−1_)
	*V*_2_	∇*f*(*x*_*k*_)	
	*V*_3_	α_*k*_*D*_*k*_ ∇ *f*(*x*_*k*_)	
	*V*_4_	${\tilde{\alpha }}_{k}D_{k}\nabla f\left(x_{k}\right)$
	*V*_5_	*x*_*k*_ − α_*k*_*D*_*k*_ ∇ *f*(*x*_*k*_)	Gradient descent within line 6 of Algorithm 1
	*V*_6_	$x_{k}-{\tilde{\alpha }}_{k}D_{k}\nabla f\left(x_{k}\right)$	
	*M*_1_	*V*_3_ − *V*_1_	
	*M*_2_	*x*_*k*_ − *x*_*k*−1_
	*M*_3_	*M*_1_ + *M*_2_	Equivalent to *v*_*k*_ within line 11 of Algorithm 1
	*M*_4_	*y*_*k*_*p*__ + *M*_3_	Equivalent to point D, which is *y*_*k*_

### 3.3 The improved step-size selection rule

In Algorithm 1, line 6 shows the proximal scaled gradient descent process under projection. Denote *t*_*k*_ = α_*k*_*D*_*k*_, which governs descent distance and affects the convergence rate during the solving process. In M-PSGP, we are committed to enhancing the selection strategy for the descent parameter *t*_*k*_. The confirmation of *D*_*k*_ in M-PSGP corresponds to the rule (Equation 13) in SGP, which implies that an innovative *t*_*k*_ is contingent on a novel α_*k*_ strategy. In M-PSGP, tailored to the *l*_1_-regularized problem (Equation 7), we propose a step-size rule BB2^*^ and a related SS^*^ rule to determine α_*k*_, meanwhile, the descent parameter *t*_*k*_ is modified. As mentioned by Crisci et al. (2024), we consider the following partition of the index set during the iteration:


$$\begin{array}{l} \begin{array}{c} J_{k}=\left\{i:x_{i}^{k}=0\wedge -\nabla f\left(x_{i}^{k}\right)\in \partial \left(\lambda {\left||\cdot |\right|}_{1}\right)\left(x_{i}^{k}\right)\right\}\\ =\left\{i:x_{i}^{k}=0\wedge -\nabla f\left(x_{i}^{k}\right)\in \left[-\lambda ,\lambda \right]\right\},\\ I_{k}=\left\{1,2,3,\ldots ,n\right\}\backslash J_{k}, \end{array} \end{array}$$

where *i* denotes the index of the zero component in *x*_*k*_. By invoking this partition in Algorithm 1, the iteration is carried out:


$$\begin{array}{l} \begin{array}{c} x_{i}^{k+1}=\{\begin{array}{cc} 0, & i\in J_{k},\\ x_{i}^{k}-\beta^{w_{k}}\alpha_{k}\left[\nabla f\left(x_{i}^{k}\right)+\text{sign}\left(t_{k}\right)\cdot \lambda \right], & i\in I_{k}, \end{array} \end{array} \end{array}$$

in which *t*_*k*_ = *x*_*k*_ − α_*k*_*D*_*k*_ ∇ *f*(*x*_*k*_). Naturally, the vector *s*_*k*_ can be separated as:


$$\begin{array}{l} \begin{array}{c} s_{k}=\left[\begin{array}{c} s_{J_{k}}\\ s_{I_{k}} \end{array}\right]=\left[\begin{array}{c} 0\\ -\beta^{w_{k}}\alpha_{k}\left[\nabla f\left(x_{i}^{k}\right)+\text{sign}\left(t_{k}\right)\cdot \lambda \right] \end{array}\right], \end{array} \end{array}$$ (20)

and the difference between the corresponding gradients *z*_*k*_ can be reformulated as:


$$\begin{array}{l} \begin{array}{c} z_{k}=\left[\begin{array}{c} z_{J_{k}}\\ z_{I_{k}} \end{array}\right]=\left[\begin{array}{c} A_{J_{k}I_{k}}s_{I_{k}}\\ A_{I_{k}I_{k}}s_{I_{k}} \end{array}\right]. \end{array} \end{array}$$ (21)

Applying Equations 20, 21 to the BB rules (Equation 14) and (Equation 15), we can obtain the following:


$$\begin{array}{l} \alpha_{k}^{\text{BB}1}=\frac{s_{I_{k-1}}^{\text{T}}D_{k}^{-1}D_{k}^{-1}s_{I_{k-1}}}{s_{I_{k-1}}^{\text{T}}D_{k}^{-1}z_{I_{k-1}}}, \end{array}$$ (22)

$$\begin{array}{l} \alpha_{k}^{\text{BB}2}=\frac{s_{I_{k-1}}^{\text{T}}D_{k}z_{I_{k-1}}}{z_{I_{k-1}}^{\text{T}}D_{k}D_{k}z_{I_{k-1}}+z_{J_{k-1}}^{\text{T}}D_{k}D_{k}z_{J_{k-1}}}. \end{array}$$ (23)

Inspired by the BB2-like rule for box-constrained problems proposed by Crisci et al. (2019), we develop a new variant, which is termed the BB2^*^ rule, as follows:


$$\begin{array}{l} \begin{array}{c} \alpha_{k}^{\text{BB}2^{*}}=\frac{s_{I_{k-1}}^{\text{T}}D_{k}z_{I_{k-1}}}{z_{I_{k-1}}^{\text{T}}D_{k}D_{k}z_{I_{k-1}}}. \end{array} \end{array}$$ (24)

Intuitively, the optimization process is accelerated by preemptively eliminating the optimal zero components, as the reciprocal of 1/α_*k*_ only depends on non-zero components. By excluding these zero components, the step-size calculation can focus on the active subspace, thereby improving the accuracy of curvature estimation, reducing redundant projections, and ultimately enhancing convergence efficiency. Furthermore, motivated by the SGP algorithm, which alternatively chooses α_*k*_ to balance convergence acceleration with algorithmic robustness, we determine α_*k*_ by switching between $\alpha_{k}^{\text{BB}2^{*}}$ and $\alpha_{k}^{\text{BB}1}$ adaptively. Thereafter, the comprehensive step-size selection rule, which we termed the SS^*^ rule, is defined as follows:


$$\begin{array}{l} \alpha_{k}=\{\begin{array}{lll} \alpha_{k}^{\text{BB}1}, & if & \frac{\alpha_{k}^{\text{BB}1}}{\alpha_{k}^{\text{BB}2^{*}}}<\tau ,\\ \alpha_{k}^{\text{BB}2^{*}}, & if & \frac{\alpha_{k}^{\text{BB}1}}{\alpha_{k}^{\text{BB}2^{*}}}\geq \tau , \end{array} \end{array}$$ (25)

where τ is the threshold determining the translation.

It is remarked that, since we propose a larger step-size rule BB2^*^ (Equation 24) than BB2 (Equation 15), it is possible that the *y*_*k*_*p*__ after gradient descent and projection yields an opposite increase. In this situation, a monotone line search, which enforces the sequence *f*(*x*_*k*_) to be strictly decreasing, might lead to multiple computation loops during backtracking. That results in a dissipation of the acceleration effect achieved by our BB2^*^ rule. Thus, equipping a non-monotone line search procedure is significant for the M-PSGP algorithm to adapt to the inherent non-monotonic nature of our step-size rules.

## 4 Computational complexity analysis and convergence analysis

In this section, we will focus on the computational complexity analysis and convergence analysis for the M-PSGP algorithm.

### 4.1 Computational complexity analysis

In our image deblurring problem, the dimension of the optimization variable is *n* = *N* = 65, 536 for 256 × 256 images. The point spread function (PSF) matrix *A* is typically a block circulant matrix with circulant blocks (BCCB). For such structured matrices, the matrix-vector product *Ax* can be computed with O(*n*log*n*) complexity by employing the fast Fourier transform (FFT) (Davis, 1979). Thus, the computational cost of major operations in one iteration of our M-PSGP algorithm is analyzed as follows:

- Gradient computation (*T*_∇_): Computing $\nabla f\left(x_{k}\right)=A^{⊤}\left(Ax_{k}-y\right)$ requires two FFT-based operations, yielding O(*n*log*n*) complexity.
- Function evaluation (*T*_*f*_): Evaluating $f\left(x_{k}\right)=\frac{1}{2}||Ax_{k}-y|{|}^{2}$ also requires FFT operations, with O(*n*log*n*) complexity.
- BB step-size computation (*T*_BB_): Calculating α_*k*_ involves vector differences and inner products, requiring O(*n*) operations.
- Diagonal scaling (*T*_*D*_): Applying the diagonal scaling matrix *D*_*k*_ to vectors is an element-wise operation with O(*n*) complexity.
- Proximal/projection operations (*T*_proj_): Both the proximal operator for the ℓ_1_-norm and the projection onto the non-negative orthant are element-wise operations with O(*n*) complexity.
- Backtracking evaluations (*N*_bt_): The average number of function evaluations during backtracking line search.
- Proximal calls (*C*_prox_): The number of proximal/projection calls per iteration (typically *C*_prox_ = 2 in M-PSGP).

The overall per-iteration computational complexity can be expressed as follows:


$$\begin{array}{l} T_{\text{iter}}=T_{\nabla }+T_{\text{BB}}+T_{D}+C_{\text{prox}}T_{\text{proj}}+N_{\text{bt}}T_{f}+O\left(n\right) \end{array}$$ (26)

Substituting the respective complexities:


$$\begin{array}{l} T_{\text{iter}}=O\left(n\log n\right)+O\left(n\right)+O\left(n\right)+O\left(n\right)+O\left(n\log n\right)+O\left(n\right)\\ =O\left(n\log n\right) \end{array}$$ (27)

Therefore, the per-iteration time complexity of the proposed M-PSGP algorithm is:


$$\begin{array}{l} \begin{array}{c} T_{\text{iter}}=O(n\log n) \end{array} \end{array}$$ (28)

The O(*n*log*n*) term dominates due to the FFT-based gradient and function evaluations, while all other operations maintain linear O(*n*) complexity. This complexity is significantly more efficient than the O(*n*^2^) complexity required by direct matrix operations, making our algorithm suitable for high-dimensional image processing problems.

### 4.2 Convergence analysis

In this section, we will focus on the convergence analysis for the M-PSGP algorithm. The main convergence guarantee of M-PSGP in the case of heavy-ball momentum acceleration is presented in Theorem 1, and its proof is based on a series of basic properties that we state in the following lemmas.

Lemma 4. Assume that *y*_*k*_ ≠ *x*_*k*_, then, the *d*_*k*_ = *y*_*k*_ − *x*_*k*_ is a strict descent direction for the function *f* at *x*_*k*_, that is, $\nabla f{\left(x_{k}\right)}^{\text{T}}d_{k}<0$.

*Proof*. The proof of this lemma is detailed in Appendix A.     □

Lemma 5. Define *e*_*k*_ = *m*_*k*_ − *x*_*k*_, where *m*_*k*_ = *x*_*k*_ − *sα*_*k*_*D*_*k*_ ∇ *f*(*x*_*k*_), it has:


$$\begin{array}{l} ||e_{k}||\leq s\alpha_{\text{max}}LG, \end{array}$$ (29)

for ||*D*_*k*_|| ≤ *L*, α_*k*_ ∈ [α_min_, α_max_] and *G* = max_*x* ∈ S_||∇*f*(*x*)||.

*Proof*. The proof of this lemma is detailed in Appendix B.

Lemma 6. Consider a Lyapunov function ${\text{Ψ}}_{k}=F\left(x\right)+\frac{\rho }{2\alpha_{k}}||e_{k}|{|}^{2}$, where *F*(*x*) is constructed as Equation 7 and ||*e*_*k*_|| is defined as (29). There exist constants σ, ρ > 0 such that:


$$\begin{array}{l} {\text{Ψ}}_{k+1}\leq {\text{Ψ}}_{k}-\sigma \beta^{w_{k}}|\nabla f{\left(x_{k}\right)}^{\text{T}}d_{k}|,k=1,2,\ldots \end{array}$$

*Proof*. The proof of this lemma is detailed in Appendix C.

Lemma 7. Let β ∈ (0, 1) and θ ∈ (0, 1) be fixed constants. For any iteration *k*, the nonmonotone backtracking line search terminates in a finite number of trials; that is, there exists *w*_*k*_ > 0 such that the following inequality holds:


$$\begin{array}{l} f\left(x_{k}+\beta^{w_{k}}d_{k}\right)\leq f_{\text{max}}+\theta \beta^{w_{k}}\nabla f{\left(x_{k}\right)}^{\text{T}}d_{k}, \end{array}$$

where *f*_max_ = max_0 ≤ *j* ≤ min(*k, M*−1)_*f*(*x*_*k*−*j*_).

*Proof*. The proof of this lemma is detailed in Appendix D.

Theorem 1. Consider the composite convex minimization problem (Equation 7) and suppose the sequence {*x*_*k*_} is generated by the M-PSGP algorithm under the assumption that the level set {*x*:*F*(*x*) ≤ *F*(*x*_0_)} is bounded. Then the sequence {*x*_*k*_} converges to a stationary point x^*^ of *F* over S = {*x* : *x* ≥ 0}, i.e.,


$$\begin{array}{l} \underset{k\to \infty }{\lim}x_{k}=x^{*}. \end{array}$$

*Proof*. The proof of this theorem is detailed in Appendix E.

## 5 Results

In this section, we conduct simulation experiments to confirm the superiority of the M-PSGP algorithm in solving *l*_1_-regularized optimization problems, with a focus on its application to image deblurring. All algorithms are implemented in Python 3.8.10, and the experiments are conducted on a computer equipped with a 12th Gen Intel(R) Core(TM) processor at 2.30 GHz.

First, we compare the performance of different BB-like step-size rules within the framework of the SGP algorithm, demonstrating the superiority of SS^*^ rules in accelerating the convergence rate and enhancing deblurring effectiveness. For the sake of convenience in notation, we name the method that incorporates SS^*^ into SGP as the SGP* algorithm. Then, we combine the unified momentum framework UM with the SGP* algorithm, which serves as the M-PSGP algorithm. The comparison between the SGP* and M-PSGP demonstrates the notable advantage of momentum acceleration. Moreover, we compare M-PSGP with other seminal methods and present its excellence.

### 5.1 Experiment settings and performance measures

**Figure 3:**
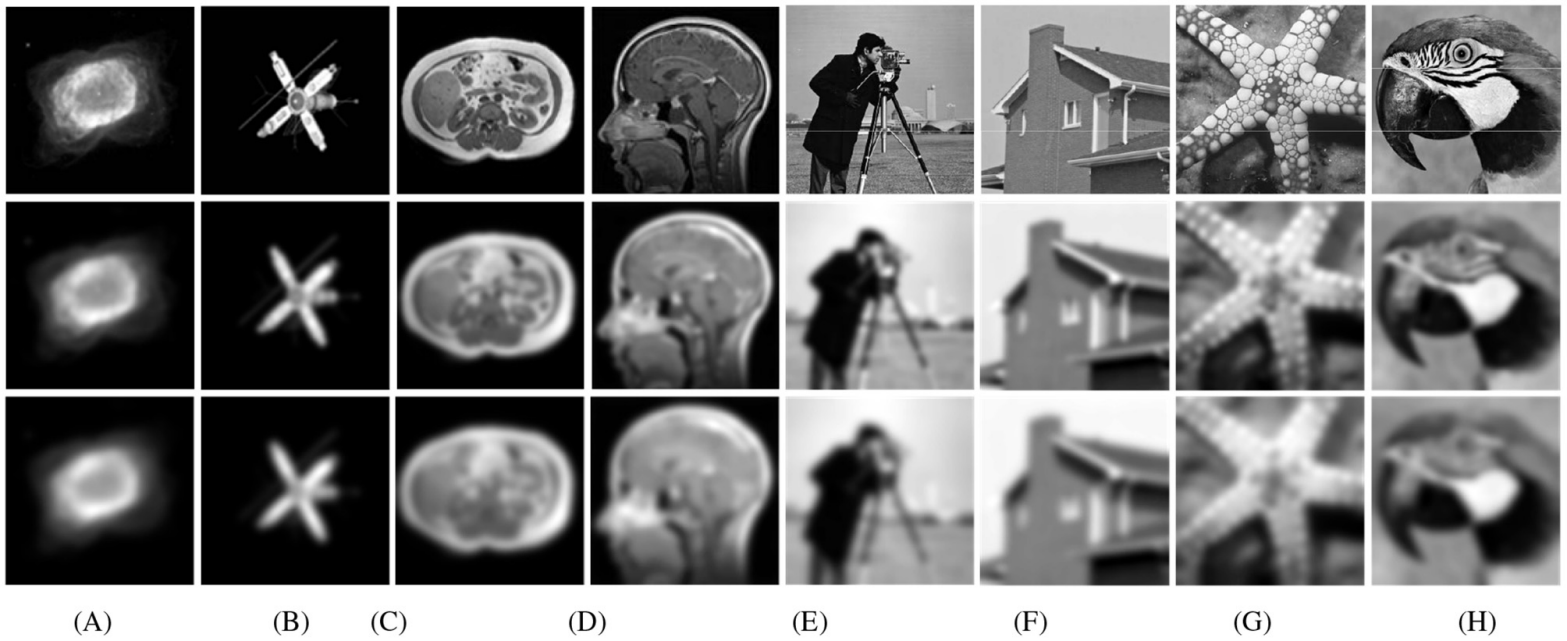
Test examples. The first row displays the original images, the second row displays the deblurred images with **σ^2^ = 5**, the third row displays the deblurred images with **σ^2^ = 10**. The test images are sequentially labeled as: **(A)** NGC7027; **(B)** SATELLITE; **(C)** CHAOS; **(D)** OASIS; **(E)** CAMERAMAN; **(F)** HOUSE; **(G)** FISHSTAR; **(H)** PARROT.

All of the algorithms are tested on problem (Equation 7). The test examples are generated by convolving the original 256 × 256 images with the Gaussian kernels of different noise levels (σ^2^ = 5 and σ^2^ = 10; higher σ values result in stronger blurring effects), followed by additive white noise with a Gaussian distribution (σ^2^ = 0.01). To ensure an objective and consistent evaluation of the proposed M-PSGP algorithm and its comparison with the baseline SGP method, we adopted the test examples used in Bonettini and Prato (2015); Federica et al. (2015) and selected two representative CT images. Furthermore, to comprehensively evaluate the generalization capability and robustness of the M-PSGP algorithm, we also selected four images of different types from the standard image processing benchmark dataset Set12 proposed by Zhang et al. (2017) as supplementary test examples. The complete set of selected test examples is presented in Figure 3.

To evaluate and compare the effectiveness of different algorithms, we measure the number of convergence iterations (ITER), the corresponding runtime (TIME) to achieve convergence, relative reconstruction error (RRE) (Bonettini et al., 2008), peak signal-to-noise ratio (PSNR) (Liao et al., 2025), and structural similarity (SSIM) (Liao et al., 2024). These indicators are defined as follows:


$$\begin{array}{l} \text{RRE}=\frac{\left||\hat{x}-x|\right|}{\left||x|\right|},\\ \text{PSNR}=10log_{10}\frac{255^{2}\times M\times N}{\sum_{m=1}^{M}\sum_{n=1}^{N}{\left(x_{m,n}-{\hat{x}}_{m,n}\right)}^{2}},\\ \text{SSIM}=\frac{\left(2\mu_{x}\mu_{\hat{x}}+c_{1}\right)\left(2\sigma_{x\hat{x}}+c_{2}\right)}{\left(\mu_{x}^{2}+\mu_{\hat{x}}^{2}+c_{1}\right)\left(\sigma_{x}^{2}+\sigma_{\hat{x}}^{2}+c_{2}\right)}, \end{array}$$

where *x* is the original image and $\hat{x}$ is the deblurred image, *M* and *N* denote the sizes along the two dimensions of the image. μ_*x*_ and $\mu_{\hat{x}}$ are the pixel-wise averages, σ_*x*_ and $\sigma_{\hat{x}}$ are the variances of *x* and $\hat{x}$, $\sigma_{x\hat{x}}$ is the covariance between *x* and $\hat{x}$, and *c*_1_ and *c*_2_ are stability constants.

In all simulations, we set the maximum number of iterations as 200, $\alpha_{\min}=10^{-10}$, $\alpha_{\max}=10^{5}$, and α_0_ = 1.3. The step size threshold τ is set as 0.15. In the backtracking loop step, the line search parameters are θ = 10^−4^, β = 0.95. The initial iteration value *x*_0_ is set as follows: $x_{i}^{0}=\frac{\overset{N}{\underset{i=1}{\sum }}y_{i}}{N}\left(i=1,2,3,\cdots \;,N\right),$ where *y* are the observed values.

### 5.2 Comparison of step-size rules in SGP

In this subsection, we inspect the performance in practical implementations of those different step-size rules:

• BB1: $\alpha_{k}=\alpha_{k}^{\text{BB}1}$, where $\alpha_{k}^{\text{BB}1}$ is defined as the rule (Equation 14).

• BB2: $\alpha_{k}=\alpha_{k}^{\text{BB}2}$, where $\alpha_{k}^{\text{BB}2}$ is defined as the rule (Equation 15).

• SS: α_*k*_ is defined following the SS algorithm proposed by Bonettini and Prato (2015).

• BB2^*^: α_*k*_ is defined following the rule (Equation 24).

• SS^*^: α_*k*_ is defined following the rule (Equation 25).

Here, the BB2^*^ and SS^*^ are our proposed rules. To adhere to the principle of a single variable, thereby enhancing the referential value of the experimental outcomes, we uniformly implement these rules in the SGP algorithm.

**Figure 4:**
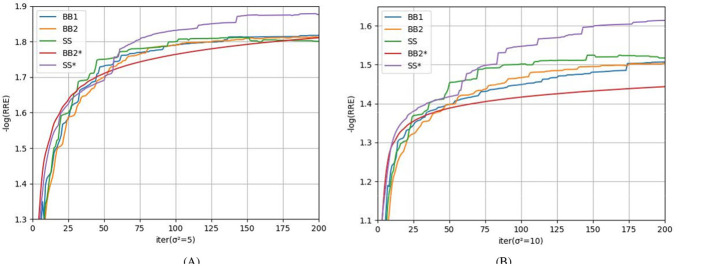
Comparison of different step-size rules in terms of the average negative logarithm of RRE: **(A)** **σ^2^ = 5**; **(B)** **σ^2^ = 10**.

The numerical results are reported in Table 2 and Figure 4. Table 2 shows that for all test images, SS^*^ could achieve the highest PSNR and SSIM on all test images, indicating the best optimization effect and the highest quality of the reconstructed images. As shown in the curve in Figure 4, it can be concluded that SS^*^ and BB2^*^ require fewer iterations to achieve the same effect compared to other rules at the preliminary iteration stage, which means that the SS^*^ and BB2^*^ rules can improve the convergence rate more than other rules. Across the comprehensive optimization stages, SS^*^ achieves the lowest RRE (the highest negative logarithm of RRE), demonstrating the dual advantages of SS^*^ in accelerating convergence while enhancing optimization accuracy. Indeed, the SGP algorithm employing the SS* method is equivalent to the M-PSGP algorithm operating without its momentum acceleration, which can be denoted as the SGP* algorithm.

**Table 2:** Performance comparison of different step-size rules under various noise levels (**σ^2^ = 5** and **σ^2^ = 10**).

**Kernel**	**Image**	**BB1**	**BB2**	**SS**	BB2^*^(proposed)	SS^*^(proposed)
σ^2^ = 5	NGC7027	39.5780/0.9634	39.5803/0.9636	39.5579/0.9634	39.8836/0.9666	**39.9833**/**0.9667**
	SATELLITE	33.6064/0.9757	33.5219/0.9755	33.6351/0.9756	32.3182/0.9683	**33.9139**/**0.9779**
	CHAOS	28.4492/0.8815	28.5246/0.8827	28.5318/0.8838	28.8542/0.8891	**29.1115**/**0.8895**
	OASIS	25.7603/0.7782	25.7585/0.7789	25.7785/0.7797	26.1355/0.7995	**26.2549**/**0.7944**
	CAMERAMAN	26.7746/0.8681	26.7028/0.8577	26.8309/0.8716	26.7797/0.8703	**26.8613**/**0.8724**
	HOUSE	29.6711/0.8774	29.8932/0.9007	30.0078/0.9092	29.9713/0.9063	**30.0477**/**0.9092**
	FISHSTAR	27.0041/0.8665	27.1139/0.8797	27.3877/0.8884	27.2619/0.8873	**27.4539**/**0.8898**
	PARROT	26.8160/0.8595	26.9666/0.8644	27.2669/0.8859	27.3357/0.8905	**27.5730**/**0.8973**
	AVERAGE	29.7075/0.8838	29.7577/0.8879	29.8746/0.8944	29.8175/0.8972	**30.1499**/**0.8996**
σ^2^ = 10	NGC7027	35.9593/0.9334	35.8644/0.9335	36.0525/0.9331	35.9286/0.9362	**36.4075**/**0.9384**
	SATELLITE	28.4599/0.9369	28.5127/0.9378	29.2375/0.9435	27.1484/0.9246	**29.8386**/**0.9492**
	CHAOS	24.5731/0.7908	24.5733/0.7917	24.5444/0.7899	24.5858/0.7919	**25.0174**/**0.8045**
	OASIS	22.5697/0.6574	22.5661/0.6566	22.5823/0.6572	22.3894/0.6547	**23.1401**/**0.6831**
	CAMERAMAN	24.1789/0.7862	24.1274/0.7836	24.4084/0.8006	24.2899/0.7987	**24.4311**/**0.8011**
	HOUSE	27.1311/0.8328	27.1667/0.8411	27.2124/0.8541	27.2322/0.8545	**27.2617**/**0.8566**
	FISHSTAR	24.5393/0.7998	24.5570/0.8001	24.7344/**0.8026**	24.6588/0.8003	**24.7516**/0.8019
	PARROT	23.9755/0.8067	24.1777/0.8198	24.3802/**0.8223**	24.2395/0.8211	**24.3888**/0.8221
	AVERAGE	26.4234/0.8180	26.4432/0.8205	26.6440/0.8254	26.3091/0.8228	**26.9046**/**0.8318**

Values indicate PSNR (dB)/SSIM metrics. Bold values represent the best performance metric in each row.

### 5.3 Comparison between SGP* and M-PSGP

**Figure 5:**
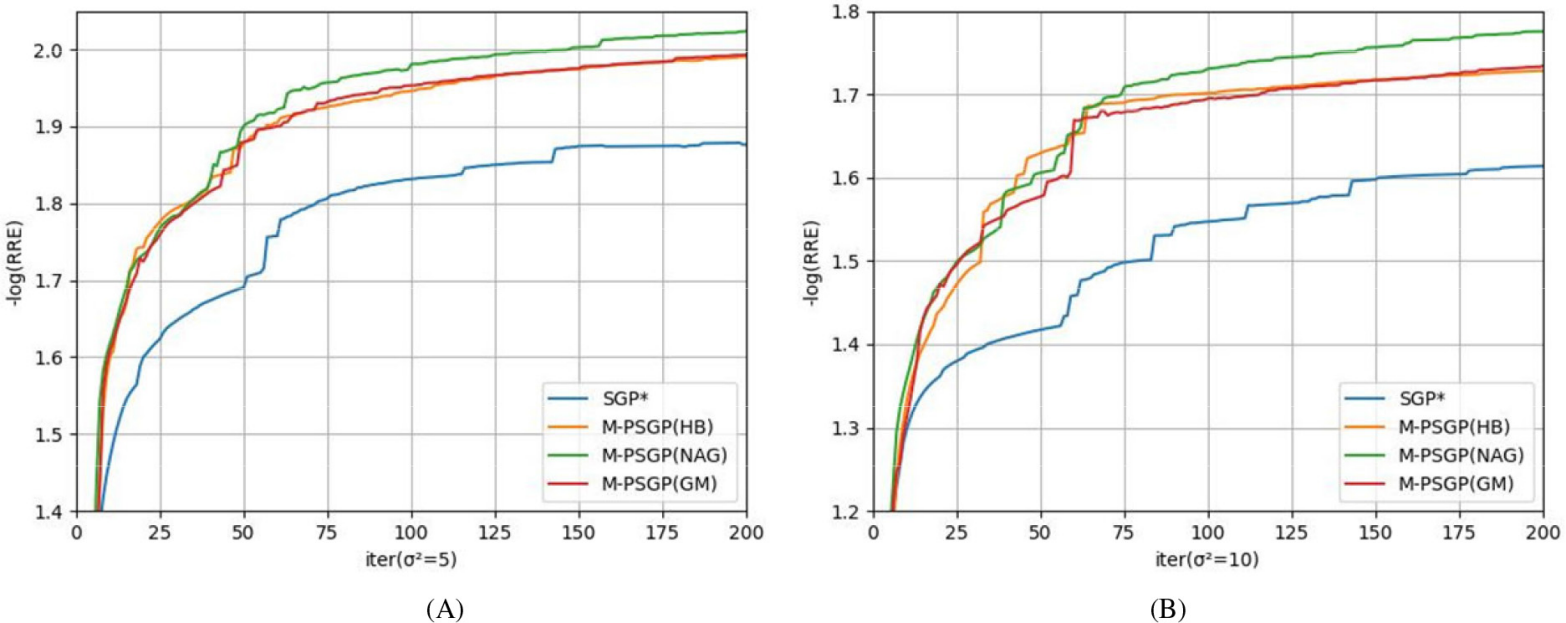
Comparison between SGP* and M-PSGP accelerated variants in terms of the average negative logarithm of RRE: **(A)** **σ^2^ = 5**; **(B)** **σ^2^ = 10**.

**Figure 6:**
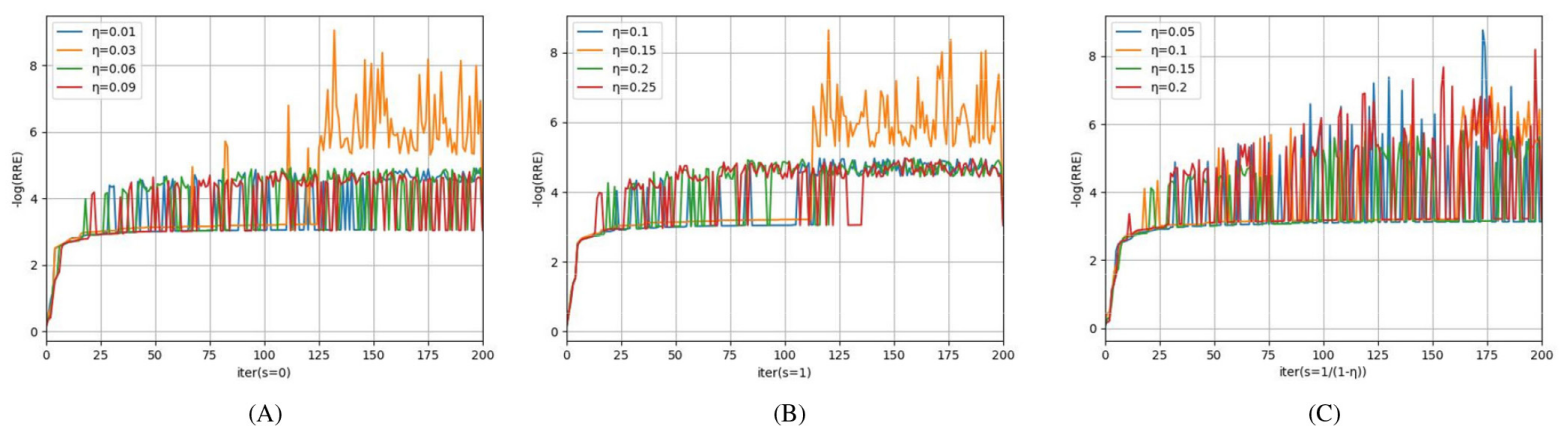
Sensitivity and optimal choice of η for M-PSGP accelerated variants: **(A)** *s* = 0; **(B)** *s* = 1; **(C)** *s* = 1/(1 − η).

Aiming to study the acceleration and stability effects of the unified momentum framework UM (Equation 16), we compare the M-PSGP algorithm with the SGP^*^ algorithm (M-PSGP without its momentum acceleration) in this subsection. Table 3 presents the numerical performance of SGP^*^ and M-PSGP variants (accelerated by HB, NAG, and GM). The results demonstrate that all M-PSGP variants can produce more efficient results than SGP^*^. From Figure 5, we can observe that the M-PSGP variants consistently outperform SGP^*^ in convergence speed across noise levels, achieving faster RRE reduction and earlier stabilization. Furthermore, as shown in Equation 16, the selection of parameter η must satisfy the range required for the convergence of the algorithms, and then within this range, values that yield higher performance metrics are chosen. To demonstrate the sensitivity of η, using the NGC7027 case with a blur level of σ^2^ = 5 as an example, we investigated the performance of three accelerated variants (HB: *s* = 0, NAG: *s* = 1, and GM: *s* = 1/1 − η) under different values of the parameter η. Using the NGC7027 case with a blur level of σ^2^ = 5 as an example. The experimental results are presented in Figure 6.

**Table 3:** Performance comparison between SGP* and M-PSGP accelerated variants under different noise levels (**σ^2^ = 5** and **σ^2^ = 10**).

**Kernel**	**Image**	SGP^*^	**M-PSGP accelerated by**		
			**Equation** **17**	**Equation** **18**	**Equation** **19**
σ^2^ = 5	NGC7027	39.9833/0.9666	40.7471/0.9695	**40.8651**/**0.9699**	40.6911/0.9694
	SATELLITE	33.9139/0.9779	34.2553/0.9779	**34.3848**/**0.9783**	34.3649/0.9783
	CHAOS	29.1115/0.8895	29.3828/0.8952	**29.8529**/**0.9041**	29.3901/0.8974
	OASIS	26.2549/0.7944	26.5816/0.8141	**26.8532**/**0.8192**	26.5982/0.8142
	CAMERAMAN	26.8613/0.8724	26.8762/0.8737	**27.0218**/**0.8776**	26.9117/0.8759
	HOUSE	30.0477/0.9092	29.6217/0.9013	30.0248/0.9054	**30.0971**/**0.9107**
	FISHSTAR	27.4539/0.8898	27.4828/0.8932	**27.5456**/**0.8960**	27.4743/0.8927
	PARROT	27.5730/0.8973	27.5137/0.8897	27.6549/0.8922	**27.6753**/**0.9079**
	AVERAGE	30.1499/0.8996	30.3077/0.9018	**30.5003**/0.9053	30.4003/**0.9058**
σ^2^ = 10	NGC7027	36.4075/0.9384	37.6049/0.9498	**37.9150**/**0.9524**	37.5544/0.9493
	SATELLITE	29.8386/0.9492	30.1958/0.9429	**31.0540**/**0.9553**	30.4011/0.9471
	CHAOS	25.0174/0.8045	26.3179/0.8388	**26.5665**/**0.8484**	26.2568/0.8371
	OASIS	23.1401/0.6831	24.3091/0.7237	**24.3651**/**0.7297**	24.3245/0.7251
	CAMERAMAN	24.4311/0.8011	24.4412/0.8010	**24.4501**/**0.8013**	24.4497/0.8011
	HOUSE	27.2617/0.8566	27.2637/0.8473	**27.2783**/**0.8573**	27.2668/0.8566
	FISHSTAR	24.7516/0.8019	24.7716/0.7994	**24.8385**/**0.8022**	24.7722/0.8007
	PARROT	24.3888/0.8211	24.3907/0.8219	**24.3952**/**0.8223**	24.3933/0.8220
	AVERAGE	26.9046/0.8318	27.4119/0.8406	**27.5996**/**0.8459**	27.4356/0.8426

Values represent PSNR(dB)/SSIM metrics. Bold values represent the best performance metric in each row.

### 5.4 Comparison of M-PSGP with other algorithms

**Figure 7:**
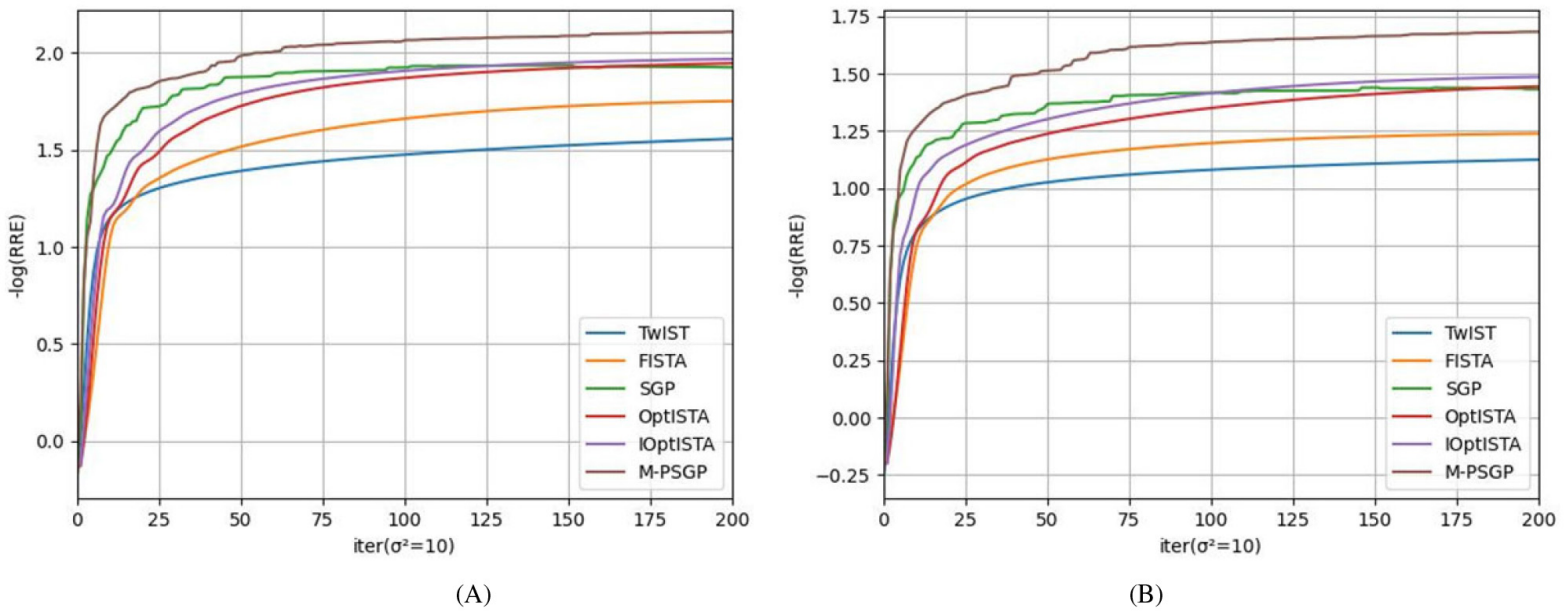
Comparison of TwIST, FISTA, SGP, OptISTA, IoptISTA and M-PSGP in terms of the average negative logarithm of RRE: **(A)** **σ^2^ = 5**; **(B)** **σ^2^ = 10**.

**Figure 8:**
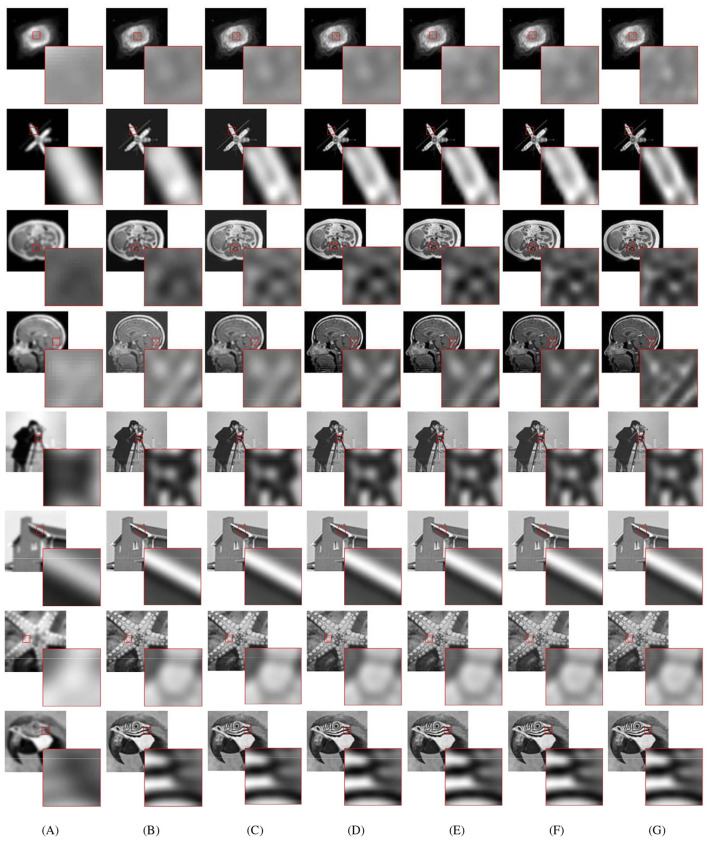
Visual reconstructed results from blurred test images (**σ^2^ = 5**). The images shown from left to right are **(A)** blurred images; **(B)** TWIST-deblurred images; **(C)** FISTA-deblurred images; **(D)** SGP-deblurred images; **(E)** OptISTA-deblurred images; **(F)** IoptISTA-deblurred images; **(G)** M-PSGP-deblurred images.

In this subsection, we present the comparison of the M-PSGP algorithm with other algorithms, including TwIST (Bioucas-Dias and Figueiredo, 2007), FISTA (Beck and Teboulle, 2009), SGP (Bonettini and Prato, 2015), OptISTA (Jang et al., 2025), and IOptISTA (Wang et al., 2025). Table 4 presents the performance metrics of M-PSGP and other seminal algorithms, from which we can conclude that M-PSGP achieves the highest PSNR and SSIM, among others. On 12 out of the 16 test examples, the M-PSGP algorithm achieved satisfactory deblurring performance in less time, which is attributed to its requiring fewer iterations to converge. On the remaining four test images, M-PSGP achieved better deblurring performance under the same iteration limit, albeit at the expense of a longer total runtime. In Figure 7, based on the average variations in curvature depicted, we further substantiate that the M-PSGP algorithm can accelerate convergence during the optimization process more than others. In Figure 8, we present a magnified view of the deblurring effects on test images under the Gaussian noise level σ^2^ = 5, which allows for a visual assessment of the M-PSGP and other algorithms. The magnified details demonstrate the superior capabilities of the M-PSGP algorithm in enhancing the overall effect of image deblurring, as well as in preserving and clarifying edges.

**Table 4:** Performance comparison of different deblurring algorithms under various noise levels (**σ^2^ = 5** and **σ^2^ = 10**).

**Kernel**	**Image**	**TWIST**	**FISTA**	**SGP**	**OptISTA**	**IOptISTA**	**M-PSGP(proposed)**
σ^2^ = 5	NCC7027	37.5447/0.9509	39.0224/0.9535	39.5579/0.9635	39.6975/0.9674	39.7114/0.9675	**40.8651**/**0.9699**
		**22.8753**/200	23.6571/200	24.1953/176	23.3126/200	23.2349/189	23.2139/**167**
	SATELLITE	28.1473/0.8976	30.7777/0.9076	33.6350/0.9756	33.8119/0.9696	33.8430/0.9707	**34.3848**/**0.9783**
		19.5327/200	17.3891/200	15.2328/161	16.6894/200	13.9163/175	**13.3951**/**148**
	CHAOS	25.8712/0.7922	27.5537/0.8005	28.5318/0.8813	28.8154/0.8804	29.0347/0.8828	**29.8529**/**0.9041**
		29.5632/200	27.6831/200	29.3262/200	28.2222/200	28.2871/200	**26.3988**/**141**
	OASIS	24.2528/0.7117	24.9531/0.7138	25.7785/0.7797	25.8660/0.7714	25.9432/0.7736	**26.8532**/**0.8192**
		31.9157/200	28.3624/200	27.4646/200	26.8934/200	27.3651/200	**26.9111**/**200**
	Cameraman	25.6017/0.8469	25.7443/0.8475	26.8309/0.8716	26.8351/**0.8787**	**27.1974**/0.8779	27.0218/0.8776
		34.3028/200	32.9612/200	31.9408/200	33.7145/200	31.9979/200	32.7244/200
	House	29.6338/0.8949	29.9337/0.8949	30.0078/0.9092	30.0529/0.9089	30.0771/0.9092	**30.0971**/**0.9107**
		41.3221/200	37.7947/200	38.4411/200	38.2826/186	33.6389/169	**21.2737**/**133**
	Fishstar	26.7017/0.8447	26.7476/0.8589	27.3877/0.8884	27.3610/0.8835	**27.6281**/**0.8555**	27.5456/0.8960
		37.6243/200	35.3721/200	20.0208/107	34.7623/200	33.6731/200	**10.5272**/**63**
	Parrot	25.7248/0.8767	26.4034/0.8782	27.2669/0.8859	27.5565/0.9046	27.6496/0.9068	**27.6753**/**0.9079**
		37.6236/200	35.3711/200	20.0290/107	34.7622/200	33.6775/200	**10.5209**/**63**
	Average	27.9347/0.8520	28.8920/0.8569	29.8746/0.8944	29.9995/0.8956	30.1356/0.8930	**30.5369**/**0.9079**
		31.84/200	29.8200/200	25.8300/169	29.5819/88	28.2200/192	**20.2502**/**139**
σ^2^ = 10	NCC7027	33.8812/0.9037	35.2076/0.9101	36.0525/0.9330	35.8559/0.9279	36.2379/0.9286	**37.9150**/**0.9524**
		19.9156/200	20.0935/200	16.3111/135	18.9412/187	17.8764/200	**13.4333**/**127**
	SATELLITE	25.2859/0.8261	26.0682/0.8248	29.2375/0.9435	29.5298/0.9414	29.8351/0.9433	**31.0540**/**0.9553**
		23.0627/200	21.3631/200	17.3982/183	18.9888/200	16.4399/154	**16.0073**/**148**
	CHAOS	22.8515/0.6288	23.3274/0.6434	24.5444/0.7899	24.7078/0.7876	24.8516/0.7946	**26.5665**/**0.8484**
		**26.5537**/198	27.5318/**185**	28.8154/192	28.0347/190	28.0712/195	26.8712/195
	OASIS	20.9931/0.5423	21.6196/0.5688	22.5824/0.6573	22.6105/0.6839	22.7438/0.6953	**24.3651**/**0.7297**
		25.2528/200	20.9531/117	16.7785/88	20.8660/125	20.7432/122	**12.8532**/**49**
	Cameraman	23.9589/0.7946	24.0975/0.7866	24.4084/0.8006	24.4903/0.8121	**24.7036**/**0.8224**	24.4501/0.8013
		14.0721/89	**9.8957**/**30**	20.9984/146	26.7152/181	23.9727/179	18.7213/66
	House	27.0941/0.8446	27.2009/0.8530	27.2124/0.8541	27.3413/0.8492	**27.3900**/**0.8512**	27.2783/0.8573
		20.1911/200	18.2147/200	20.4413/121	18.0324/186	23.6301/147	**8.4733**/**27**
	Fishstar	24.5846/0.7616	24.6578/0.7784	24.7344/0.8026	25.2344/0.8127	**25.2718**/**0.8236**	24.8385/0.8022
		33.9719/200	34.7324/200	11.5346/63	21.7623/146	15.6743/111	**7.3867**/**41**
	Parrot	23.6300/0.8137	23.8572/0.8148	24.3802/0.8223	24.0451/0.8129	24.1471/0.8153	**24.3952**/**0.8223**
		40.2161/200	36.9713/200	22.4794/87	36.3971/200	32.1775/200	**7.9909**/**44**
	Average	25.2849/0.7644	25.7545/0.7725	26.6440/0.8254	26.7269/0.8285	26.8976/0.8343	**27.6078**/**0.8461**
		25.4/186	23.7195/167	19.3446/127	23.7171/156	22.3262/164	**13.9672**/**87**

Values indicate PSNR(dB)/SSIM/RUN-TIME(s) metrics. Bold values represent the best performance metric in each row.

## 6 Conclusion and discussion

In this study, we propose the M-PSGP algorithm and demonstrate its significant improvement in optimizing *l*_1_-regularized problems, with applications to image deblurring. The integration of the improved step-size rule BB2^*^ and the unified momentum framework UM has led to accelerated convergence rates and improved performance in image deblurring tasks. Numerical experiments have demonstrated that the M-PSGP algorithm outperforms existing proximal gradient projection algorithms, TwIST, FISTA, SGP, OptISTA, and IOptISTA. The M-PSGP algorithm has presented a credible alternative to conventional techniques in image deblurring and has demonstrated potential applications in other domains, where *l*_1_-regularized optimization problems are prevalent. In the future, we will endeavor to apply the M-PSGP algorithm to other optimization problems, such as ridge regression and non-convex optimization, thereby enhancing its applicability to a broader range of signal processing tasks. Furthermore, with the potential to provide novel perspectives for tackling large-scale optimization problems, the application of the M-PSGP algorithm to parameter training in machine learning is also a significant research topic.

While this study presents a promising optimization framework, its practical deployment may face certain limitations that warrant further discussion. First, the performance of the method depends on the selection of several hyperparameters (e.g., η). Although the algorithm incorporates adaptive mechanisms, its sensitivity to these settings may present challenges in practical applications, where parameter tuning is limited or robustness is essential. Second, the convergence analysis and numerical experiments are conducted under standard assumptions. The method's performance in non-Gaussian or heavy-tailed noise environments remains an open question. Such conditions are common in real-world data (e.g., sensor data), and the robustness of momentum acceleration and line search strategies in these settings warrants further investigation. Acknowledging these aspects would provide a more comprehensive view of the method's operational scope and guide future research toward enhancing its practical robustness and applicability.

## Data Availability

The original contributions presented in the study are included in the article/Supplementary material, further inquiries can be directed to the corresponding author.
