# Supplementary material for: M-PSGP: a momentum-based proximal scaled gradient projection algorithm for nonsmooth optimization with application to image deblurring
Source: Front Big Data. 2025 Nov 24;8:1704189. doi: 10.3389/fdata.2025.1704189 (PMC12682648; doi:10.3389/fdata.2025.1704189)
Supplement: Supplementary file 1 [file Presentation_1.pdf]

A PROOF OF LEMMA 4

PROOF. Following the line 6 of Algorithm 1 and considering the first-order stationarity condition of the proximal operator, it follows:

$$\frac{1}{\alpha_k} D_k^{-1}(x_k - p_k) - \nabla f(x_k) \in \partial g(p_k).$$

By Definition 2, there exists $w_k \in \partial g(p_k)$ such that

$$\frac{1}{\alpha_k} D_k^{-1}(x_k - p_k) - \nabla f(x_k) = w_k. \tag{1}$$

From the Definition 4 and Lemma 2, it can be obtained that, for $\forall y \in \mathcal{S}$, the $\mathbf{\Pi}_{\mathcal{S},D}$ satisfies

$$(\mathbf{\Pi}_{\mathcal{S},D}(x) - x)^{\mathrm{T}} D^{-1} (\mathbf{\Pi}_{\mathcal{S},D}(x) - y)) \leq 0,$$

and invoking line 7 of Algorithm 1, we can get:

$$(y_{k_p} - p_k)^{\mathrm{T}} D_k^{-1} (y_{k_p} - x_k) \leq 0.$$

Denoting $\bar{d}_k = y_{k_p} - x_k$, then we obtain:

$$(\bar{d}_k + x_k - p_k)^{\mathrm{T}} D_k^{-1} \bar{d}_k \leq 0, \text{ i.e., } \bar{d}_k^{\mathrm{T}} D_k^{-1} \bar{d}_k \leq (p_k - x_k)^{\mathrm{T}} D_k^{-1} \bar{d}_k,$$

sequentially,

$$\frac{1}{\alpha_k}(x_k - p_k)^{\mathrm{T}} D_k^{-1} \bar{d}_k \leq -\frac{1}{\alpha_k} \bar{d}_k^{\mathrm{T}} D_k^{-1} \bar{d}_k. \tag{2}$$

By substituting (2) into (1), we derive:

$$(\nabla f(x_k) + w_k)^{\mathrm{T}} \bar{d}_k \leq -\frac{1}{\alpha_k} \bar{d}_k^{\mathrm{T}} D_k^{-1} \bar{d}_k \leq 0,$$

i.e.

$$\nabla f(x_k)^{\mathrm{T}} \bar{d}_k \leq (\nabla f(x_k) + w_k)^{\mathrm{T}} \bar{d}_k \leq -\frac{1}{\alpha_k} \bar{d}_k^{\mathrm{T}} D_k^{-1} \bar{d}_k \leq 0. \tag{3}$$

As $d_k = \bar{d}_k + \eta v_k$ in Algorithm 1, ηv_k can be denoted as $\tilde{d}_k$. Denoting $z_k = m_k - m_{k-1}$, the momentum item in line 11 can be rewritten as:

$$\tilde{p_k} = \mathrm{prox}_{\alpha_k g}^{D_k^{-1}}(z_k), \tag{4}$$

$$v_k = \mathbf{\Pi}_{\mathcal{S},D_k}(\tilde{p_k}). \tag{5}$$

Following (4) and considering the first-order stationarity condition of the proximal operator, it can be derived that:

$$\frac{1}{\alpha_k} D_k^{-1}(z_k - \tilde{p_k}) \in \partial g(\tilde{p_k}), \tag{6}$$

i.e., there is $\tilde{w_k} \in \partial g(\tilde{p_k})$ such that

$$\frac{1}{\alpha_k} D_k^{-1}(z_k - \tilde{p_k}) = \tilde{w_k}.$$

In problem (7), function g is defined as $\lambda\|x\|^2$ and the feasible set is $\mathcal{S} = \{x : x \geq 0\}$, which means $w \in \partial g(x)$ such that $w > 0$ for any $x \in \mathcal{S}$. As well as the projection operator $\mathbf{\Pi}_{\mathcal{S},D}$ produces a nonnegative vector v_k, it has $\tilde{w_k} v_k \geq 0$ during the iteration. Thus, from (6), we can derive:

$$(\tilde{p_k} - z_k)^{\mathrm{T}} D_k^{-1} v_k \leq 0. \tag{7}$$

Following (5), we can obtain:

$$(v_k - \tilde{p_k})^{\mathrm{T}} D_k^{-1}(v_k - x_k) \leq 0, \quad \forall x_k \in \mathcal{S}.$$

Then, it satisfies:

$$(v_k - \tilde{p_k})^{\mathrm{T}} D_k^{-1} v_k \leq 0, \tag{8}$$

The superposition of (7) and (8) leads to:

$$(v_k - z_k)^{\mathrm{T}} D_k^{-1} v_k \leq 0,$$

i.e.

$$z_k^{\mathrm{T}} D_k^{-1} v_k \geq v_k^{\mathrm{T}} D_k^{-1} v_k.$$

During the momentum acceleration in Algorithm 1, two distinct scenarios arise that $s = 0$ and $s > 0$. In what follows, we will analyze the case $s = 0$. The analysis for $s > 0$ will be investigated in our future work.

In the case that $s = 0$, $z_k = x_k - x_{k-1} = \beta^{w_{k-1}} d_{k-1}$, which denotes z_k already lies in the feasible cone of the proximal operation. Thus,

$$\tilde{d_k} = \eta v_k = \eta \beta^{w_{k-1}} d_{k-1}.$$

When $k = 1$, $m_0 = m_1 = x_1$, then $v_1 = 0$, $\nabla f(x_1)^{\mathrm{T}} d_1 = \nabla f(x_1)^{\mathrm{T}} \bar{d_1} \leq 0$. When $k = 2$, $\nabla f(x_2)^{\mathrm{T}} \tilde{d_2} = \eta \beta^{w_1} \nabla f(x_2)^{\mathrm{T}} d_1 \leq 0$. Then, under the assumption that there exists a $k > 2$ satisfies $\nabla f(x_k)^{\mathrm{T}} \tilde{d_k} \leq 0$, we can derive:

$$\nabla f(x_{k+1})^{\mathrm{T}} \tilde{d}_{k+1} = \eta \beta^{w_k} \nabla f(x_k)^{\mathrm{T}} d_k = \eta \beta^{w_k} \nabla f(x_k)^{\mathrm{T}} (\bar{d_k} + \tilde{d_k}) \leq 0.$$

Hence, through mathematical induction, we can conclude:

$$\nabla f(x_k) \tilde{d_k} \leq 0, \quad \text{when } s = 0. \tag{9}$$

The superposition of (3) and (9) leads to:

$$\nabla f(x_k)^{\mathrm{T}} (\bar{d_k} + \tilde{d_k}) \leq 0, \text{ i.e., } \nabla f(x_k)^{\mathrm{T}} d_k \leq 0.$$

Since $y_k \neq x_k$, $d_k \neq 0$ and we final obtain:

$$\nabla f(x_k)^{\mathrm{T}} d_k < 0.$$

B PROOF OF LEMMA 5

PROOF. We have

$$e_k = -s\alpha_k D_k \nabla f(x_k),$$

Since $\|D_k\| \leq L$, $\alpha_k \leq \alpha_{\max}$ and $\|\nabla f(x)\| \leq \max_{x \in \mathcal{S}} \|\nabla f(x)\| = G$, it has:

$$\|e_k\| = s\alpha_k \|D_k \nabla f(x_k)\| \leq s\alpha_{\max} LG. \tag{10}$$

Remark that when $s = 0$, $m_k = x_k$ and $e_k = m_k - x_k = 0$, which satisfies (10).

C PROOF OF LEMMA 6

PROOF. According to the Armijo condition line19 during the backtracking loop of Algorithm 1, it can be concluded that

$$f(x_{k+1}) \leq f_{\max} + \theta \beta^{w_k} \nabla f(x_k)^{\mathrm{T}} d_k,$$

where $f_{\max} = \max_{0 \leq j \leq \min(k, M-1)} f(x_{k-j})$. It has been proven that the nonmonotone Armijo condition maintains almost sure convergence to critical points with a dynamically adjusted window size M (Zhang and Hager, 2004). Here, we set $M = 1$ for simplicity and we can derive $f_{\max} = f(x_k)$, hence,

$$f(x_{k+1}) - f(x_k) \leq \theta \beta^{w_k} \nabla f(x_k)^{\mathrm{T}} d_k.$$

Assume $\Delta x = x_{k+1} - x_k = \beta^{w_k} d_k$, then, $g(x_{k+1}) - g(x_k) = g(x_k + \triangle x) - g(x_k)$. As d_k is a strict descent, $\Delta x \to O(\beta^{w_k})$, it obtains $g(x_{k+1}) - g(x_k) \to O(\beta^{w_k})$ and

$$F(x_{k+1}) - F(x_k) \leq -\theta \beta^{w_k} \left| \nabla f(x_k)^{\mathrm{T}} d_k \right|,$$

Following (10),

$$\|e_{k+1}\|^2 - \|e_k\|^2 = s^2 \left(\alpha_{k+1}^2 \|D_{k+1} \nabla f(x_{k+1})\|^2 - \alpha_k^2 \|D_k \nabla f(x_k)\|^2 \right).$$

When $s = 0$, $\|e_{k+1}\|^2 - \|e_k\|^2 = 0$ and $\Psi(k+1) - \Psi(k) = F(x_{k+1}) - F(x_k) \leq -\theta \beta^{w_k} \left| \nabla f(x_k)^{\mathrm{T}} d_k \right|$ for $\sigma = \theta > 0$.

D PROOF OF LEMMA 7

PROOF. Denote $t = \beta^{w_k}$. Since ∇f is L-Lipschitz continuous (Equation 8), for any $t > 0$, it has:

$$f(x_k + t d_k) \leq f(x_k) + t \nabla f(x_k)^{\mathrm{T}} d_k + \frac{L}{2} t^2 \|d_k\|^2,$$

then,

$$f(x_k + td_k) \leq f_{\max} + t\nabla f(x_k)^{\mathrm{T}} d_k + \frac{L}{2} t^2 \|d_k\|^2. \tag{11}$$

The line search condition requires:

$$f(x_k + td_k) \leq f_{\max} + \theta t \nabla f(x_k)^{\mathrm{T}} d_k. \tag{12}$$

The subtraction of (12) from (11) gives:

$$(1-\theta) t |\nabla f(x_k)^{\mathrm{T}} d_k| \geq \frac{L}{2} t^2 \|d_k\|^2,$$

then,

$$t \leq \frac{2(1-\theta)|\nabla f(x_k)^{\mathrm{T}} d_k|}{L\|d_k\|^2}.$$

As $t = \beta^{w_k}$, we derive:

$$\beta^{w_k} \leq \frac{2(1-\theta)|\nabla f(x_k)^{\mathrm{T}} d_k|}{L\|d_k\|^2}, \text{ i.e., } w_k \leq \frac{\ln\left(\frac{2(1-\theta)|\nabla f(x_k)^{\mathrm{T}} d_k|}{L\|d_k\|^2}\right)}{\ln \beta}.$$

For any $\theta \in (1 - \frac{L\|d_k\|^2}{2|\nabla f(x_k)^{\mathrm{T}} d_k|}, 1)$ and $\beta \in (0,1)$, $w_k > 0$ is guaranteed. Hence, there exists a finite integer w_k that satisfies the Amijo condition.

E PROOF OF THEOREM 1

PROOF. Define the Lyapunov function:

$$\Psi_k := F(x_k) + \frac{\rho}{2\alpha_k}\|e_k\|^2, \quad \rho > 0.$$

Under assumption that F is bounded, and given the boundedness of $\|e_k\|^2$ as shown in Lemma 5, we have $\Psi_k \geq F_{\inf}$ and Ψ_k is bounded. By Lemma 6, there exists $\sigma > 0$ such that

$$\Psi_{k+1} \leq \Psi_k - \sigma \beta^{w_k} |\nabla f(x_k)^{\mathrm{T}} d_k|,$$

which implies Ψ_k is monotonically decreasing and convergent. Summing both sides gives a telescoping sum:

$$\sum_{k=1}^{\infty} \sigma \beta^{w_k} |\nabla f(x_k)^{\mathrm{T}} d_k| \leq \Psi_1 - \lim_{k\to\infty} \Psi_k < \infty,$$

and,

$$\lim_{k\to\infty} \beta^{w_k} \left|\nabla f(x_k)^{\mathrm{T}} d_k\right| = 0.$$

By Lemma 7, $\beta^{w_k} > 0$ and w_k bounded away from 0, hence, we conclude:

$$\lim_{k\to\infty} \nabla f(x_k)^{\mathrm{T}} d_k = 0. \tag{13}$$

From (3) in Lemma 4, it can be derived that:

$$\left|\nabla f(x_k)^{\mathrm{T}} \bar{d}_k\right| \geq c_1 \|\bar{d}_k\|^2, \tag{14}$$

superposing (14) and (9) gives:

$$\left|\nabla f(x_k)^{\mathrm{T}} (\bar{d}_k + \tilde{d}_k)\right| \geq c_1 \|\bar{d}_k\|^2,$$

Since $d_k = \bar{d}_k + \tilde{d}_k$, it exists $c > 0$ such that

$$\left|\nabla f(x_k)^{\mathrm{T}} d_k\right| \geq c \|d_k\|^2.$$

Following (13), we obtain:

$$\lim_{k\to\infty} \left|\nabla f(x_k)^{\mathrm{T}} d_k\right| = 0.$$

Thus,

$$\lim_{k\to\infty} \|d_k\|^2 = 0,$$

and

$$\lim_{k\to\infty} \|d_k\| = \lim_{k\to\infty} \sqrt{\|d_k\|^2} = \sqrt{\lim_{k\to\infty} \|d_k\|^2} = 0.$$

Recall the update $x_{k+1} = x_k + \beta^{w_k} d_k$, we deduce

$$\lim_{k\to\infty} \|x_{k+1} - x_k\| = \lim_{k\to\infty} \beta^{w_k} \|d_k\| = 0.$$

Hence, $\{x_k\}$ is a Cauchy-like sequence. And we denote $\bar{x} = \lim_{k\to\infty} x_k$.

Since the proximal operator $\mathrm{prox}_{\alpha g}^{D^{-1}}$ is 1-Lipschitz continuous (Lemma 1), it has:

$$\lim_{k\to\infty} p_k = \lim_{k\to\infty} \mathrm{prox}_{\alpha_k g}^{D_k^{-1}} (x_k - \alpha_k D_k \nabla f(x_k)) = \mathrm{prox}_{\alpha_k g}^{D_k^{-1}} (\bar{x} - \alpha_k D_k \nabla f(\bar{x})).$$

As well as the projection operator $\mathbf{\Pi}_{\mathcal{S},D}(x)$ is L^2-Lipschitz continuous (Lemma 2), it obtains:

$$\lim_{k\to\infty} y_{k_p} = \lim_{k\to\infty} \mathbf{\Pi}_{\mathcal{S},D_k}(p_k) = \mathbf{\Pi}_{\mathcal{S},D_k}\left[\mathrm{prox}_{\alpha_k g}^{D_k^{-1}} (\bar{x} - \alpha_k D_k \nabla f(\bar{x}))\right].$$

Since $\lim_{k\to\infty} \|x_{k+1} - x_k\| = 0$, it can be derived that $\lim_{k\to\infty} \|m_k - m_{k-1}\| = 0$. Thus,

$$\lim_{k\to\infty} v_k = \lim_{k\to\infty} \mathbf{\Pi}_{\mathcal{S},D_k}\left[\mathrm{prox}_{\alpha_k g}^{D_k^{-1}} (m_k - m_{k-1})\right] = 0.$$

Hence,

$$\lim_{k\to\infty} y_k = \lim_{k\to\infty} y_{k_p} + \eta \lim_{k\to\infty} v_k = \mathbf{\Pi}_{\mathcal{S},D_k}\left[\mathrm{prox}_{\alpha_k g}^{D_k^{-1}}\left(\bar{x} - \alpha_k D_k \nabla f(\bar{x})\right)\right].$$

From $\lim_{k\to\infty} \|d_k\| = 0$, we can derive that $\lim_{k\to\infty} y_k = \lim_{k\to\infty}(x_k + d_k) = \lim_{k\to\infty} x_k = \bar{x}$. Then, we obtain:

$$\bar{x} = \mathbf{\Pi}_{\mathcal{S},D_k}\left[\mathrm{prox}_{\alpha_k g}^{D_k^{-1}}\left(\bar{x} - \alpha_k D_k \nabla f(\bar{x})\right)\right],$$

which is exactly equivalent to the stationarity condition of Lemma 3. Thus, $\bar{x}$ is the limited point of the sequence $\{x_k\}$, as well as a stationary point x^* of the problem (7).

REFERENCES

Zhang, H. and Hager, W. (2004). A nonmonotone line search technique and its application to unconstrained optimization. *SIAM J. Optim.* 14, 1043–1056. doi:10.1137/S1052623403428208
